# Secreted phosphoprotein 1 as a potential prognostic and immunotherapy biomarker in multiple human cancers

**DOI:** 10.1080/21655979.2021.2020391

**Published:** 2022-01-22

**Authors:** Ping Zeng, Xujun Zhang, Tianxin Xiang, Zongxin Ling, Chenhong Lin, Hongyan Diao

**Affiliations:** a State Key Laboratory for Diagnosis and Treatment of Infectious Diseases, National Clinical Research Center for Infectious Diseases, Collaborative Innovation Center for Diagnosis and Treatment of Infectious Diseases, the First Affiliated Hospital, College of Medicine, Zhejiang University, Hangzhou, China; b Department of Hospital Infection Control, The First Affiliated Hospital of Nanchang University, Nanchang, China

**Keywords:** SPP1, prognosis, immune infiltration, tumor microenvironment

## Abstract

Secreted phosphoprotein 1 (*SPP1*) is involved in immune regulation, cell survival, and tumor progression. Studies have demonstrated that *SPP1* plays an important role in certain individual tumors. However, the expression profile and oncogenic features of *SPP1* in diverse cancers are remaining unknown. Therefore, we performed a comprehensive analysis using The Cancer Genome Atlas (TCGA) database. Raw data of 33 cancer types were download from the University of California Santa Cruz (UCSC) Xena website. The expression of *SPP1* and its relationship with tumor prognosis, immune invasion, tumor microenvironment, and immunotherapy were analyzed using the R language. The function analysis was conducted using Gene Set Enrichment Analysis (GSEA). The oncogenic features of *SPP1* was validated by wound-healing assay and EdU staining assay. *SPP1* highly expressed in most cancers. The expression of *SPP1* was significant related to prognosis, tumor mutation burden (TMB), microsatellite instability (MSI), and immune checkpoint genes, suggested that *SPP1* plays an essential role in the tumor immune microenvironment and immune cell infiltration. The immune/stromal scores correlated positively with the *SPP1* expression, and the relationship was affected by tumor heterogeneity and immunotherapy. In addition, *SPP1* could predict the response of tumor immunotherapy. Functional analysis revealed the *SPP1*-associated terms and pathways. Finally, *SPP1* significantly elevated cell proliferation and migration in A549, Huh7, HT-29, A2780 tumor cell lines. In conclusion, this study indicated that *SPP1* involved in tumorigenesis, tumor progression, and regulated tumor immune microenvironment, revealing *SPP1* might be a potential target for evaluating prognosis and immunotherapy in multiple cancers.

## Introduction

Cancer is a primary cause of high morbidity and high mortality worldwide. According to the World Health Organization, More than 18 million cancer new cases and 9.6 million cancer deaths occurred in 2018 [1]. Cancer is a complex and heterogeneous disease, involving interactions between tumor and immune system, making it difficult to uncover its pathogenesis. In recent years, the pan-cancer analysis has been extensively used in the analysis and study of cancer disease [2].

The common and differential features existing in various types of cancers have been contributing to researches focusing on potential mechanisms of cancer, as well as estimating the clinical prognosis. The Cancer Genome Atlas (TCGA) is based on multiple levels of tumor data from different human cancer cell lines and tissues, focusing on molecular mutations associated with the occurrence and progression of cancer. The TCGA database contains data of 33 cancer types, which can be used for survival analysis and prognosis evaluation, such as overall survival (OS), disease-specific survival (DSS), disease-free interval (DFI), and progression-free interval (PFI). The TCGA contributed to establishing the significance of cancer genomics, helping us understanding cancer in multiple levels, and profoundly impacting the treatment concept of cancer.

As a multifunctional and secreted integrin-binding glycoprotein, secreted phosphoprotein 1 (*SPP1*) is found to express in different tissues and cell types. The biological functions of *SPP1* are variable, specifically in some certain physiological and pathological conditions, including drug resistance, cell proliferation, invasion, survival, stem-like behavior and tumor metastasis [3]. Accumulating evidences indicated that *SPP1* plays an important role in several tumor-associated processes, such as proliferation, invasion, migration, angiogenesis, and metastasis [4,5]. *SPP1* promotes epithelial-mesenchymal transition during metastasis and regulates the tumor microenvironment in favor of metastasis [6,7]. In both experimental and clinical studies, *SPP1* has been shown to correlated with the progression and prognosis of different tumors [8]. Recent researches demonstrated that overexpressing of *SPP1* could be used as an indicator of poor prognosis in numerous human cancers, for instance, lung cancer, gastric carcinoma, colorectal cancer, and prostate cancers [8]. Studies have shown that *SPP1* regulates tumorigenesis by interacting with integrins or the CD44 receptor, subsequently activating Wnt signaling [9] or focal adhesion kinase (FAK)/P-glycoprotein (P-GP) signaling transduction pathways [10].

In the present study, we aimed to explore the *SPP1* expression profiles and oncogenic features among human cancers using the TCGA project for comprehensive analysis. Our findings suggested that *SPP1* is a potential target for evaluating patient prognosis and immunotherapy in multiple human cancers.

## Materials and Methods

### TGCA data acquisition and processing

As a landmark cancer genomics program, TCGA had characterized over 20,000 primary cancer and matched normal samples from 33 cancer types. Transcriptome RNA-seq data of 33 cancers were download from the University of California Santa Cruz (UCSC) Xena website (http://xena.ucsc.edu/) [11]. 33 cancer types were included: adrenocortical carcinoma (ACC), bladder Urothelial Carcinoma (BLCA), breast invasive carcinoma (BRCA), cervical squamous cell carcinoma and endocervical adenocarcinoma (CESC), cholangiocarcinoma (CHOL), colon adenocarcinoma (COAD), Lymphoid Neoplasm Diffuse Large B-cell Lymphoma (DLBC), esophageal carcinoma (ESCA), glioblastoma multiforme (GBM), head and neck squamous cell carcinoma (HNSC), kidney chromophobe (KICH), kidney renal clear cell carcinoma (KIRC), kidney renal papillary cell carcinoma (KIRP), acute myeloid leukemia (LAML), brain lower grade glioma (LGG), liver hepatocellular carcinoma (LIHC), lung adenocarcinoma (LUAD), lung squamous cell carcinoma (LUSC), mesothelioma (MESO), ovarian serous cystadenocarcinoma (OV), pancreatic adenocarcinoma (PAAD), pheochromocytoma and paraganglioma (PCPG), prostate adenocarcinoma (PRAD), rectum adenocarcinoma (READ), sarcoma (SARC), skin cutaneous melanoma (SKCM), stomach adenocarcinoma (STAD), testicular germ cell tumors (TGCT), thyroid carcinoma (THCA), thymoma (THYM), uterine corpus endometrial carcinoma (UCEC), uterine carcinosarcoma (UCS), and uveal melanoma (UVM).

### *Analysis of* SPP1 *expression in cancers*

The Ensembl gene ID of transcriptome data was converted into Symbol ID to extract the expression of target genes in each tumor patient. Differential gene expression was analyzed based on ggpubr R package (https://www.rdocumentation.org/packages/ggpubr) in R software (version 3.6.3, 29 February 2020, R Foundation for Statistical Computing, Vienna, Austria). In addition, the gene expression was also identified via the Oncomine database (http://www.oncomine.org/resource/login.html) [12]. Setting the significant threshold: P-value of 0.001, fold change of 2, and gene ranking of 10%.

### Signature score calculation

The signature score reflected the status of the studies biological process in a tumor and were calculated according to the method of Rocha et al [13]. Cutoff-high (50%) and cutoff-low (50%) values were used to split the high-expression and low-expression cohorts, patients were divided into two groups. The difference of groups was tested by Wilcoxon signed-rank, using Benjamini and Hochberg correction for multiple testing within each database.

### Survival analysis and relationship with clinical stage

The survival database of 33 cancer types (survival status, time, and tumor stage) were download from UCSC Xena database. To assess the correlation between gene expression and prognosis of cancers, Kaplan-Meier (KM) curves and Cox proportional hazard regression survival analyses were performed using the survival package (https://CRAN.R-project.org/package=survival) [14]. The survival analysis included OS, DSS, DFI, PFI. Cutoff-high (50%) and cutoff-low (50%) values were used as the expression thresholds for splitting the high-expression and low-expression cohorts, patients were divided into two groups. In addition, we also analyzed the clinical correlation between gene expression and pathological stage, histological grade, age, and gender.

### TMB and MSI analysis

TMB is the total number of mutations identified in per tumor sample [15]. We download the mutation data from UCSC Xena database, the correlation between gene expression and the TMB level is analyzed by spearman correlation. Statistical analysis and corresponding visualization were performed using the R software. MSI is due to the nucleotide insertions or deletions in tumor cells of the microsatellite loci changes in the length of repetitive microsatellite sequences [16]. The correlation between gene expression and the MSI level is also analyzed by spearman correlation.

### Tumor microenvironment

We used estimate package (https://R-Forge.R-project.org/projects/estimate/) to calculate the tumor microenvironment indicators for each tumor sample and obtain the immune cell score, stromal cell content, and tumor purity [14]. The correlation between gene expression and the tumor microenvironment indicators is analyzed by spearman correlation. Statistical analysis and corresponding visualization (P < 0.001) were performed using the R software.

### *Relationship between* SPP1 *expression and immunity*

To estimate relative proportion of 22 types of infiltrated immune cells in 33 cancer types, Cell-type identification by Estimating Relative Subsets of RNA Transcripts (CIBERSORT) algorithm was conducted to estimate the relationship between gene expression and 22 infiltrated immune cells based on expression file [17]. We selected the TIICs gene markers from previous research [18] and examined the association with *SPP1* using R software. Spearman’s correlation coefficient and statistical significance was visualized in heatmap.

### Prediction of immunotherapy response

Immunotherapy datasets were downloaded from the GEO database (https://www.ncbi.nlm.nih.gov/geo/) including GSE111636, GSE67501, GSE26383, GSE79691, and GSE100797. These cohorts were used for prediction of patient response to immunotherapy [19]. The GSE111636 cohort, a BLCA cohort receiving anti-PD-L1 antibody atezolizumab immunotherapy, was also included for prediction of immunotherapy response. The GSE67501 was an RCC cohort receiving anti-PD-L1 immunotherapy, GSE26383, GSE79691, and GSE100797 were SKCM cohorts receiving same immunotherapy. The RCC cohort was a cohort receiving immune checkpoint therapy [20]. The t-test P < 0.05 was utilized to determine the statistical significance. We calculated the correlation between two variables using the Spearman method. The threshold of P < 0.05 (Spearman’s correlation test) indicates the significance of correlation. To compare the predictive power of different genomic features for immune signatures, time-dependent receiver-operating characteristic (ROC) curves analysis were performed by using SPSS version 19.0 software package. Statistical analysis was performed by using GraphPad Prism version 7.0 or SPSS version 19.0 software package. A two-tailed P < 0.05 was considered statistically significant.

### Gene set enrichment analysis

Functional analysis of gene was proceeded with GSEA [21]. We downloaded the GO (c5.all.v7.1.symboles.gml) and KEGG (c2.cp.kegg.v7.1.symboles.gml) biological process gene sets from the GSEA website (http://www.gsea-msigdb.org/gsea/index.jsp). R software was conducted to gene set enrichment analysis (P < 0.05) and exhibited the top five terms.

### *Analysis of* SPP1 *and immune-related molecules expression in liver cancers*

The concentration of *SPP1* in the liver cancer tissue were detected by commercial ELISA (Enzyme-linked immunosorbent assay) kits (MultiSciences) as specified by the manufacturer. The mRNA expression of *SPP1* and immune-related molecules were measured by quantitative real-time PCR (qRT-PCR). Total RNA was isolated using Trizol reagent (Takara). cDNA was synthesized using a PrimeScriptTM RT Msater Mis (Takara). qRT-PCR analyses were conducted with SYBR® Premix Ex TaqTM II (Takara) with specific primers as follows:

SPP1: 5ʹ-CTCCATTGACTCGAACGACTC-3ʹ (F),

5ʹ-CAGGTCTGCGAAACTTCTTAGAT-3ʹ (R);

CD44: 5ʹ-CTGCCGCTTTGCAGGTGTA-3ʹ (F),

5ʹ-CATTGTGGGCAAGGTGCTATT-3ʹ (R);

CD80: 5ʹ-AAACTCGCATCTACTGGCAAA-3ʹ (F),

5ʹ-GGTTCTTGTACTCGGGCCATA-3ʹ (R);

LAIR1: 5ʹ-ATCGGGGTCTCAGTGGTCTTC-3ʹ (F),

5ʹ-TGCTTTATCTGATTCTGGCGATG-3ʹ (R);

NRP1: 5ʹ-GGCGCTTTTCGCAACGATAAA-3ʹ (F),

5ʹ-TCGCATTTTTCACTTGGGTGAT-3ʹ (R);

HAVCR2: 5ʹ-CTGCTGCTACTACTTACAAGGTC-3ʹ (F),

5ʹ-GCAGGGCAGATAGGCATTCT-3ʹ (R).

### Immunohistochemistry

Immunohistochemistry was performed as reported elsewhere [22]. Paraffin sections of tumor and para-tumor tissues underwent dewaxing, hydration and antigen repair. Following blocking with serum, the sections were incubated with anti-SPP1 antibody (Abcam) at 4°C overnight. The slides were washed three times with PBS and incubated with a secondary antibody at 37°C for 30 min. Then, streptavidin–horseradish peroxidase was applied at 37°C for 30 min, and the slides were stained with DAB solution. Images were obtained using a direct optical microscope.

### Cell migration and cell proliferation assays

The human cell lines A549 (lung carcinoma), HuH-7 (hepatoma carcinoma) and HT-29 (colorectal carcinoma) were purchased from the Cell Bank of Chinese Academy of Sciences. The human cell lines A2780 (ovarian carcinoma) was obtained from the Tumor Cell Bank of the Chinese Academy of Medical Sciences (Beijing, China). Cell migration was determined by performing a wound healing assay. Briefly, cells were inoculated into 6-well plates. Cells reached 90% confluence and were scratched using a 100 μl pipette tip to form a wound-like gap. The cells were maintained in medium, and images were captured at 0 hour and 24 hours. ImageJ software was used to quantify the area of the wound to calculate the cell migration rate. EdU staining was used for the detection of cell proliferation. Briefly, cells were seeded in 24-well plates at a density of 2 × 10^3^ cells/well. EdU kit (Beyotime, Shanghai, China) was used for detecting cell proliferation according to the manufacturer’s instruction.

### Statistical analysis

Gene differential expression from TCGA database was analyzed by Wilcoxon rank sum test. Survival data was analyzed using the Kaplan-Meier and Cox statistical methods. Spearman correlation analysis was used for the correlation analysis in this study. The difference in *SPP1* expression between different tumor stages were compared suing the Kruskal-Wallis test. R software (version 3.6.3) was performed all analyses and chart visualize. Measurement data were expressed as the mean ± standard deviation (SD) from three independent experiments. The tumor and normal samples were compared using the unpaired t-tests. P-value <0.05 was considered statistically significant.

## Results

*SPP1*, as an important gene in certain cancer types, involved in the occurrence and development of tumors. We speculated that *SPP1* might be used as a potential prognostic and immune-related biomarker in human cancers. In this study, we conducted the correlation analysis between *SPP1* expression and clinical characterize, survival, TMB, MSI, the tumor immune microenvironment, immunotherapy, and GSEA. Furthermore, we also performed in vivo experiments to evaluate the oncogenic effect of *SPP1*. These results identified that high expression of *SPP1* could serve as a biomarker for prognostic and tumor immunotherapy. This study will contribute to a deeper understanding of the critical role of *SPP1* in human cancers.

### *Differences of* SPP1 *gene expression in human cancers*

**Figure 1.**
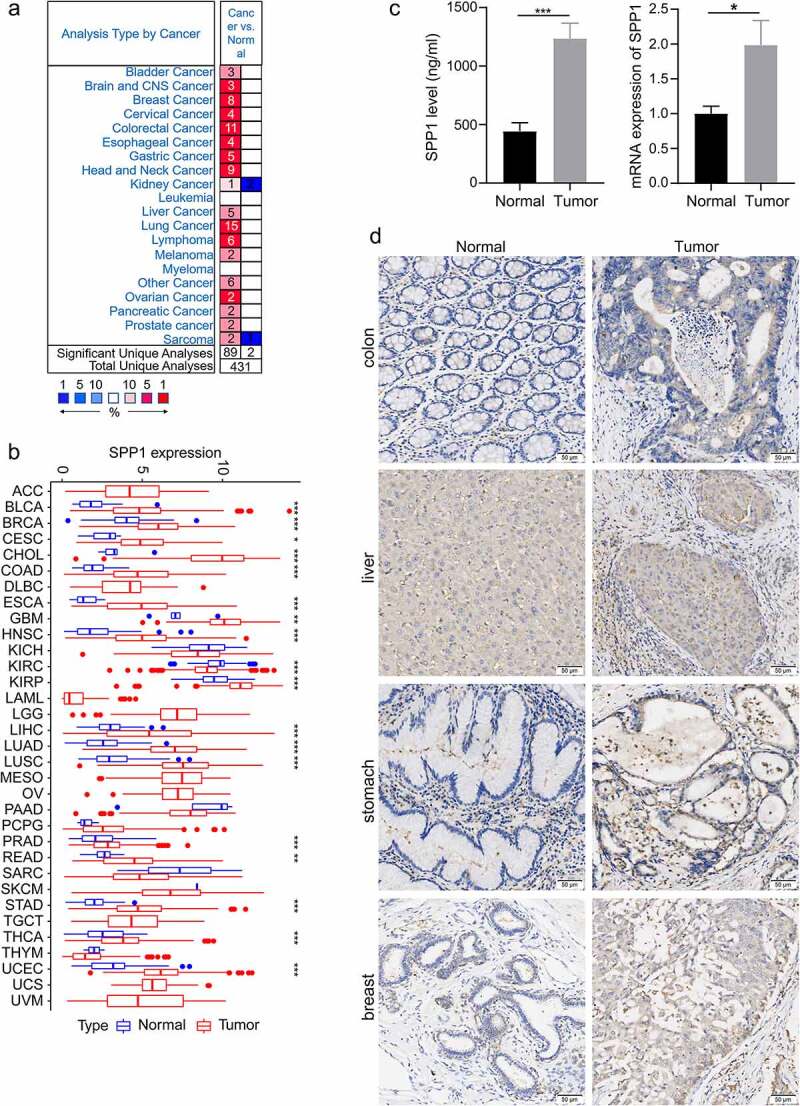
*SPP1* expression levels in different cancer types. (a) *SPP1* expression in different tumor tissues compared with that in normal tissues in the Oncomine database. Red represents the number of studies reporting elevated *SPP1* expression in tumor tissue; Blue represents the number of studies reporting elevated *SPP1* expression in normal tissue. (b) The *SPP1* expression profile in 33 cancer types from the TGCA database. (c) Protein and mRNA expression of *SPP1*. Data was expressed as mean ± SD from three independent experiments, n = 10. (d) Immunohistochemistry staining showed the expression of *SPP1* in the tumor and normal tissues. (*P < 0.05, **P < 0.01, ***P < 0.001).

In order to investigate the *SPP1* expression differences in tumor and normal samples of cancers. Initially, we used the Oncomine database to analyze the expression level of *SPP1* in different tumor and normal tissues. The result showed that *SPP1* was overexpressed in multiple types of cancer, including bladder, brain and central nervous system, breast, cervical, colorectal, esophageal, gastric, head and neck, kidney, liver, lung, ovarian, pancreatic, and prostate cancers, and in lymphoma, melanoma, and sarcoma in most data sets (Figure 1(a)). In some data sets, *SPP1* expression was lower in kidney cancer and sarcoma compared with that in normal tissues. The details information regarding the differential expression of *SPP1* in cancers versus normal tissue are summarized in Table S1. In addition, to further assess *SPP1* expression levels in human cancers, we obtained the TGCA-derived transcriptome RNA-seq data of 33 cancers from the UCSC Xena database and analyzed the *SPP1* expression using the R software. We found that *SPP1* mRNA expression was significantly higher in BLCA, BRCA, CESC, CHOL, COAD, ESCA, GBM, HNSC, KIRP, LIHC, LUAD, LUSC, PRAD, READ, STAD, THCA, and UCEC tumor tissues compared with that in normal tissues, indicating that it might play an oncogenic role in the development of most tumors. However, *SPP1* expression was lower in KIRC, KICH, PAAD, SARC, and THYM compared with that in normal tissues (Figure 1(b)). Except for that in KIRC, *SPP1* expression was not statistically significant in the other tumors. To confirm our analysis results, we used liver cancer tissues and para-cancer tissues to verify the expression of *SPP1* with ELISA and qRT-PCR experiment. These results supported that *SPP1* expression was significantly higher in liver cancer tissues compared with para-cancer tissues (Figure 1(c)). In addition, immunohistochemical assay results showed that *SPP1* highly expressed in multiple types of cancer, including breast, colon, gastric, and liver (Figure 1(d), Table S2 and S3).

### *The association of* SPP1 *expression with molecular signatures in human cancers*

In order to validate the important role of *SPP1* in human cancers, we studied some well-known biological processes including differentiation, proliferation, retinoblastoma (RB) pathways, TP53, and centrosome amplification. The most fundamental trait of tumor cells is differentiation and proliferation. We studied differentiation using nine genes (ZIC1, TCF7L1, KLF5, MYBL2, NFE2L3, TEAD4, ILF3, HMGA1, HMGB3), which overexpressed in poorly differentiated bladder, glioblastoma, and breast tumors [23]. 110 genes with predictive and prognostic effects were used to study proliferation [24]. Centrosome amplification were found in multiple cancer types and studied using a 20 genes signature (CA20) about poorly prognosis [25]. We explored DNA damage and apoptosis pathways using RB [26] and TP53 signature [27]. These scores were basically consistent with the expression trend of *SPP1* (Figure S1). In most cancers, high expression of *SPP1* means more dedifferentiation, more proliferation, more RB and TP53 mutations, and higher centrosome amplification.

### *Prognostic assessment and clinical correlation of* SPP1 *in human cancers*

**Figure 2.**
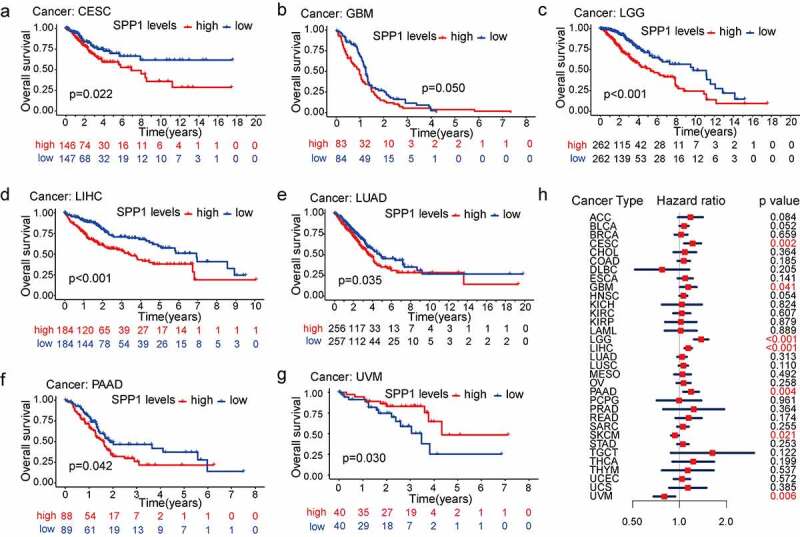
Analysis of the association between overall survival and *SPP1* expression in different cancer types. (a-g) Kaplan-Meier survival curves displaying the correlation between *SPP1* expression and overall survival. Only P < 0.05 is shown. (h) Cox analysis showing the correlation between *SPP1* expression and overall survival.

To assess the predictive value of the *SPP1* expression in the prognosis of different types of cancer, we divided all patients with cancer into high-expression and low-expression groups according to the median value of their *SPP1* expression and conducted survival analysis (OS, DSS, DFI, and PFI) using the Kaplan-Meier and COX methods. The Kaplan-Meier analysis indicated that the high *SPP1* expression significantly decreased OS in six cancers: CESC (p = 0.022), GBM (p = 0.050), LGG (P < 0.001), LIHC (P < 0.001), LUAD (p = 0.035), and PAAD (p = 0.042) (Figure 2(a-f)). High *SPP1* expression was associated with increased OS only in UVM (Figure 2(g)). In addition, we revalidated the OS analysis proposed by venet et al [28]. The results were consistent with the above analysis for four cancer types including CESC, LGG, LIHC, and UVM (Figure S2). Furthermore, we investigated the relationship between *SPP1* expression, as a continuous variable, survival time and survival status using Cox analysis. In CESC, GBM, LGG, LIHC, PAAD, SKCM, and UVM (P < 0.05), *SPP1* expression was associated with OS. The hazard ratio (HR) value was greater than 1, indicating that *SPP1* is a high-risk gene in tumors. In other words, the higher the expression of *SPP1*, the worse the prognosis (Figure 2(h)). In the DSS analysis, except in UVM, patients with high *SPP1* expression had a significantly worse prognosis in ESCA, LGG, LIHC, PAAD, and PRAD (Figure S3). In the DFI and PFI analysis, we observed the same phenomenon in certain types of cancer (Figure S4, S5). These results confirmed that *SPP1* might act as a powerful biomarker for predicting prognosis in most cancers. Increased and decreased *SPP1* expression had different prognostic values depending on the type of cancer. In addition, we investigated the relationship between *SPP1* expression and clinical characteristics (pathological stage, histological grade, age, and gender) of 33 types of cancer. Increased expression of *SPP1* was only positively correlated with certain tumor stages, such as COAD, ESCA, LIHC, and READ (Figure S6). *SPP1* expression was correlated to pathological stages including BLCA, LGG, LGG, and LIHC. The difference was statistically significant (P < 0.05) with a higher grade being associated with a higher expression level (Figure S7). Gender difference existed in most tumors, but they were not associated with the expression level of *SPP1* (Table S4). However, ESCA was more common in males, in which the expression level of *SPP1* was higher, and SARC occurred more frequently in women, in which *SPP1* expression was lower (Figure S8a, b). Age is associated closely with the occurrence of various tumors. Patients aged ≥ 60 years old formed a higher proportion in BLCA, COAD, LUAD, LUSC, and STAD. Patients aged < 60 years old formed a higher proportion in ACC, CESC, LGG, PCPG, TGCT, and THCA (Table S4). *SPP1* expression levels were markedly higher in older (≥ 60 years old) than in younger (< 60 years old) patients in BLCA, CESC, HNSC, LIHC, PRAD, SARC, and THYM. Only in SKCM was *SPP1* expression higher in younger patients (Figure S8c-j).

### *Correlation between* SPP1 *expression and gene alterations*

**Figure 3.**
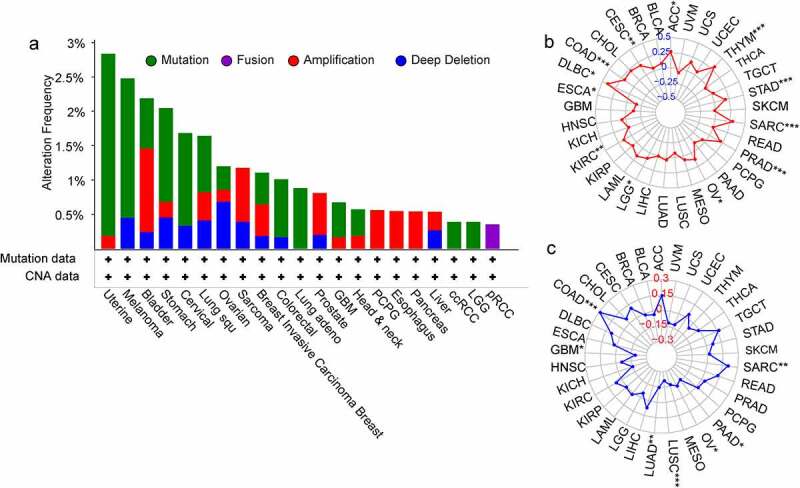
Genetic mutations of *SPP1* in different cancer types. (a) The alteration frequency with mutation type. (b) TMB radar map. (c) MSI radar map. (*P < 0.05, **P < 0.01, ***P < 0.001).

It is widely acknowledged that tumorigenesis is mainly caused by mutations in certain genes. We assessed the genetic alterations of the *SPP1* genes in patients with cancer using the cBioPortal database. The *SPP1* mutation frequency was the primary alteration in uterine tumors, melanoma, stomach tumors, cervical tumors, lung squamous cell carcinoma, colorectal tumors, lung adenocarcinoma, GBM, HNSC, ccRCC, and LGG. In bladder tumors, sarcoma, breast tumors, prostate tumors, PCPG, esophagus tumors, and pancreas tumors, gene amplification was the most important genetic alteration (Figure 3(a)). In addition, we analyzed the correlation between *SPP1* expression and the TMB and MSI (Table S5). *SPP1* expression correlated positively with the TMB in ACC, CESC, COAD, KIRC, LGG, OV, PRAD, SARC, STAD, and THYM, and correlated negatively in ESCA (Figure 3(b)). *SPP1* expression correlated positively with MSI in COAD, SARC, and correlated negatively in GBM, LUAD, LUSC, MESO, OV, and PAAD (Figure 3(c)).

### *Correlation between* SPP1 *expression and the tumor immune microenvironment*

**Figure 4.**
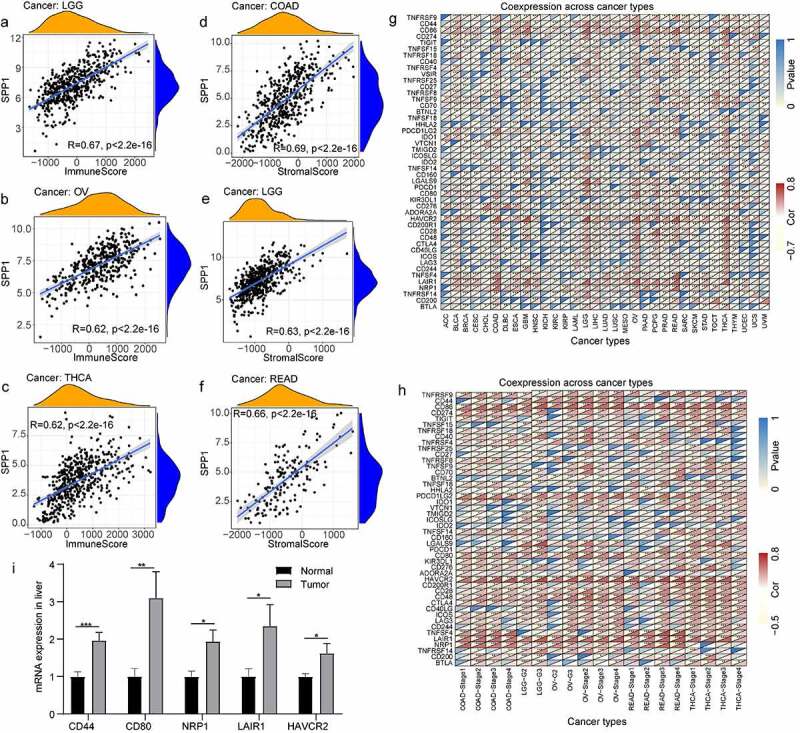
Correlation between *SPP1* expression and immunity. (a-c) Top three cancers by immune score. (d-f) Top three cancers by stromal score. (g) Heatmap of *SPP1* co-expression with immune checkpoint genes in 33 cancer types. (h) Heatmap of *SPP1* co-expression with immune checkpoint genes based on different stages and grades of the tumor. (i) The mRNA expression of CD44, CD80, HAVCR2, LAIR1, NRP1. Data was expressed as mean ± SD from three independent experiments, n = 10. (*P < 0.05, **P < 0.01, ***P < 0.001).

The tumor microenvironment is a complex milieu of stromal cells, tumor cells, and immune cells. Stromal cells support tumor growth [29], and immune cells promote or inhibit tumor growth [30]. To better understand the relevance and underlying mechanism of *SPP1* expression in cancer, we evaluated the relationship between immune and stromal scores and *SPP1* expression using the CIBERSORT analysis. *SPP1* expression correlated positively with the tumor immune and stromal scores at P < 0.001 (Table S6). The top three tumors that correlated most significantly with *SPP1* were LGG, OV, and THCA (immune score) (Figure 4(a-c)), and COAD, LGG, and READ (stromal score) (Figure 4(d-f)). We further performed a subgroup analysis of *SPP1* expression with immune and stromal scores based on different stages and grades of the tumors, including COAD, LGG, OV, READ, and THCA. *SPP1* was significantly positive correlated with immune and stromal scores in different stages of COAD and OV. In the histological grade of LGG and OV (G2 and G3), *SPP1* also showed the same trend. This result indicated that tumor grades and stages did not affect the relationship between *SPP1* and immune/stromal score. However, in READ and THCA, SPP1 was not correlated with stage І of READ and stage Ⅳ of THCA, only correlated with other stages (Table S7). This result indicated that tumor grades and stages affected the relationship between *SPP1* and immune/stromal score.

In addition, we analyzed the co-expression between *SPP1* and 47 genes associated with immune infiltrating cell types in these tumors, including those encoding the tumor necrosis factor (TNF) superfamily (TNFSF), the TNF receptor superfamily (TNFRSF), multiple CD molecules (CD40, CD44, CD86, CD27, CD70, CD160, CD80, CD28, CD48, CD244, CD274, and CD200), and markers of exhausted T cells, such as PDCD1 (programmed cell death 1) and PDCD1LG2 (programmed cell death 1 ligand 2), CTLA4 (cytotoxic T-lymphocyte associated protein 4), LAG3 (lymphocyte activating 3), and HAVCR2 (Hepatitis A virus cellular receptor 2). In most tumors, *SPP1* was co-expressed with markers of immune cells and showed significant positive correlations with them. For example, except for KIR3DL1 (encoding killer cell immunoglobulin like receptor, three Ig domains and long cytoplasmic tail 1), HHLA2 (encoding HERV-HLTR-associating 2), TNFSF9, TNFRSF25, and TNFRSF18, *SPP1* expression correlated significantly and positively with other immune cells in LGG (Figure 4(g)). In LIHC, *SPP1* was correlated with more than half of these genes including NRP1 (neuropilin 1), LAIR1 (leukocyte associated immunoglobulin like receptor 1), CTLA4, HAVCR2, and CD80. Similarly, we further performed correlation analysis of *SPP1* expression with these immune checkpoint genes based on different stages and grades of the tumor. Heatmap showing most genes positively correlated with *SPP1*, especially HAVCR2, NRP1 (neuropilin 1), LAIR1 (leukocyte associated immunoglobulin like receptor 1), CD80, PDLD1LG2, and CD86 (Figure 4(h)). *SPP1* expression was observed to correlated positively with immune checkpoint genes in multiple types of cancer. Therefore, we hypothesized that SPP1 might associate with immunotherapy. To further validate the analysis results, we examined some immune checkpoint genes expression in liver cancer. The results showed that these genes were highly expressed in liver cancer including CD80, CD44, HAVCR2, NRP1, and LAIR1 (Figure 4(i)). Together, these data indicated that high *SPP1* expression was widely associated with immunity in cancers.

**Figure 5.**
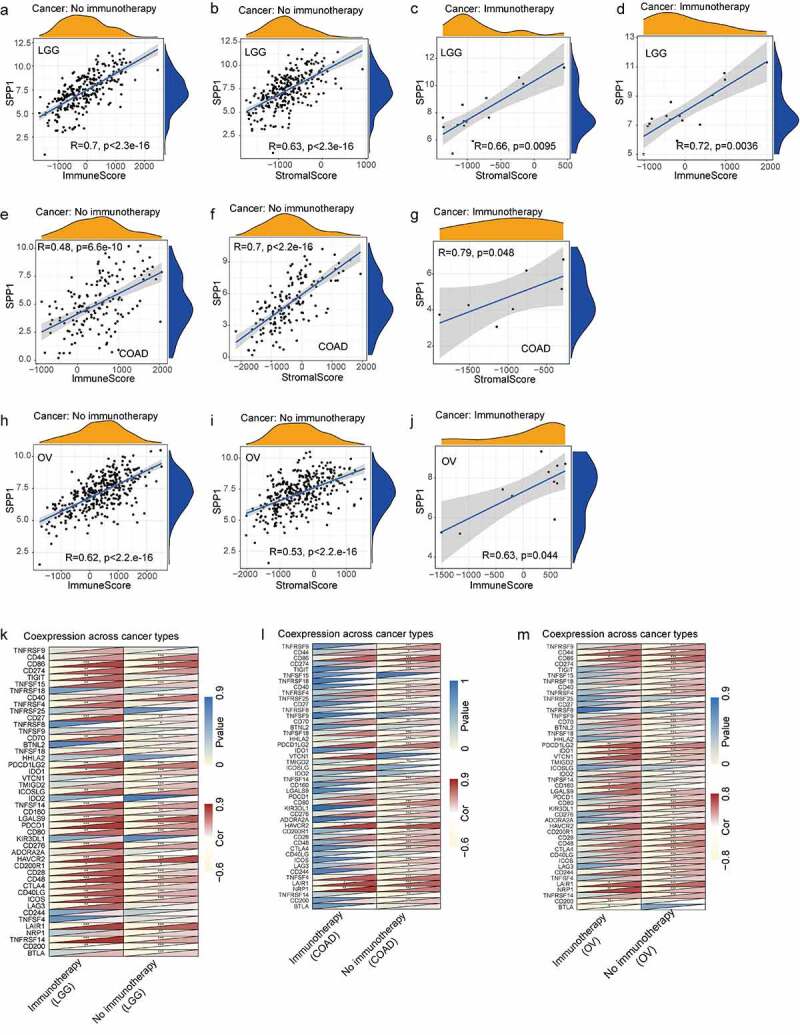
Effect of immunotherapy on the correlation between SPP1 and tumor microenvironment. (a-j) *SPP1* expression with immune and stromal scores in COAD, LGG, and OV patients treated with and without immunotherapy. (k-m) The heatmap displayed the correlation between *SPP1* and immune checkpoint genes in COAD, LGG, and OV patients treated with and without immunotherapy. (*P < 0.05, **P < 0.01, ***P < 0.001).

We performed a correlation analysis of *SPP1* expression with immune and stromal scores in COAD, LGG, and OV patients treated with or without immunotherapy. In LGG, *SPP1* was significantly positive correlated with immune and stromal scores regardless of whether patients received immunotherapy or not (Figure 5(a-d)). The heatmap also showed that *SPP1* and immune checkpoint genes were co-expression in immunotherapy and no immunotherapy group (Figure 5(k)). The results indicated that immunotherapy did not affect the correlation between *SPP1* and immune/stromal score in LGG. In COAD, *SPP1* was correlated with stromal score in immunotherapy and no immunotherapy groups, but only correlated with immune score in no immunotherapy group (Figure 5(e-g)). In OV, *SPP1* was correlated with immune score in immunotherapy and no immunotherapy groups, but only correlated with stromal score in no immunotherapy group (Figure 5(h-j)). The heatmap displayed that the expression of *SPP1* in COAD and OV was only related to immune checkpoint genes in no immunotherapy group (Figure 5(l,m)). These results indicated that immunotherapy affected immune/stromal score in COAD and OV.

Tumor-infiltrating immune cells (TIICs) are a part of the complex microenvironment that regulates tumor development and progression [31]. Therefore, we calculated the relative abundance of 22 types of immune cells in each tumor, and analyzed their correlation with *SPP1* expression. The results indicated that *SPP1* expression had significant correlations with resting mast cells in 7 types of cancer, with macrophages in 21 types of cancer, with neutrophils in 10 types of cancer, with B cell in 6 types of cancer, with DC in 5 types of cancer, with CD8 in 5 types of cancer, and with monocytes in 5 types of cancer (Table S8). The results also revealed that at the level of immune cell infiltration, BRCA, COAD, GBM, LUAD, SARC and STAD correlated most strongly with *SPP1* expression.

### *Potential of* SPP1 *as an indicator of response to immunotherapy*

**Figure 6.**
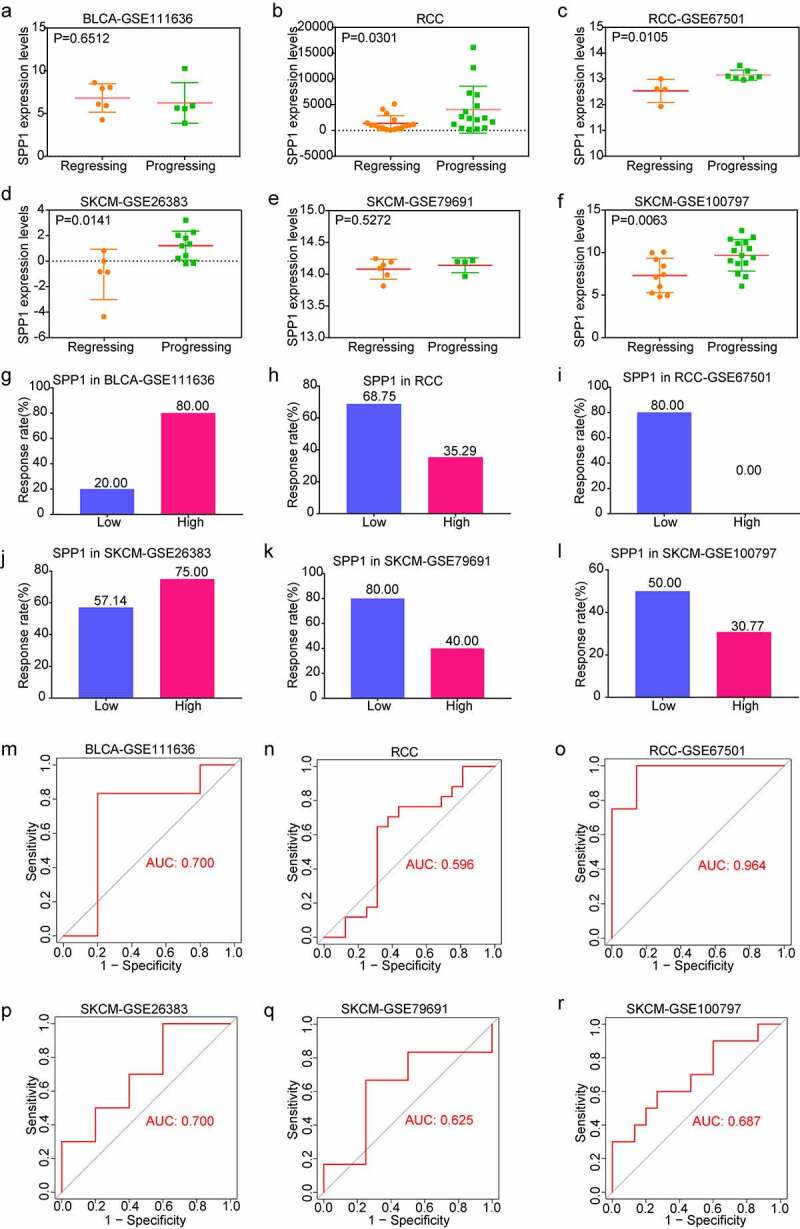
Predictive value of *SPP1* for immunotherapy. (a-f) The association of *SPP1* expression with immunotherapy regressing and progressing. (g-l) The predicted response rate of immunotherapy to anti-PD-L1 in six datasets. (m-r) ROC curves analysis of the predictive value of *SPP1* for immunotherapy in six datasets.

To investigate the role of *SPP1* in tumor immunotherapy, we downloaded and analyzed immunotherapy datasets from the GEO database including GSE111636, GSE67501, GSE26383, GSE79691, and GSE100797. The RCC cohort was derived from data which was given in the literature with receiving immune checkpoint therapy [20]. In order to analyze the relationship between *SPP1* expression and the effect of immunotherapy, we divided patients into two groups according immunotherapeutic effect. With the except of GSE111363 and GSE79691 cohorts, *SPP1* expression showed a high level in progressing group and a low level in regressing group including RCC, GSE67501, GSE26383, and GSE100797 cohorts (Figure 6(a-f)). Furthermore, on the basis of *SPP1* expression, we divided patients into two groups (low-expression and high-expression) compared the immunotherapy response rate. We found that the lower *SPP1* was related to a better response rate to immunotherapy than the high-expression group (68.75% versus 35.29% in RCC; 80% versus 0% in GSE6701; 80% versus 20% in GSE79691; 50% versus 30.77% in GSE100797) (Figure 6(h, i, k, l)). In another two cohorts, the high-expression group had a higher response rate than the low-expression group in the GSE111636 and GSE26383 cohorts (80% versus 20% in the GSE111636 cohort; 75% versus 57.14% in the GSE26383 cohort) (Figure 6(g, j)). In addition, we analyzed the predictive value of *SPP1* for immunotherapy using the ROC curve. From the value of the area under curve (AUC), *SPP1* has significant prediction power for tumor immunotherapy (AUC: 0.700 in GSE111636; AUC: 0.596 in RCC; AUC: 0.964 in GSE67501; AUC: 0.700 in GSE26383; AUC: 0.625 in GSE79691; AUC: 0.687 in GSE100797) (Figure 6(m-r)). Collectively, these results revealed that *SPP1* has the potential as a predictive indicator for the effectiveness of immunotherapy.

### Functional analysis

**Figure 7.**
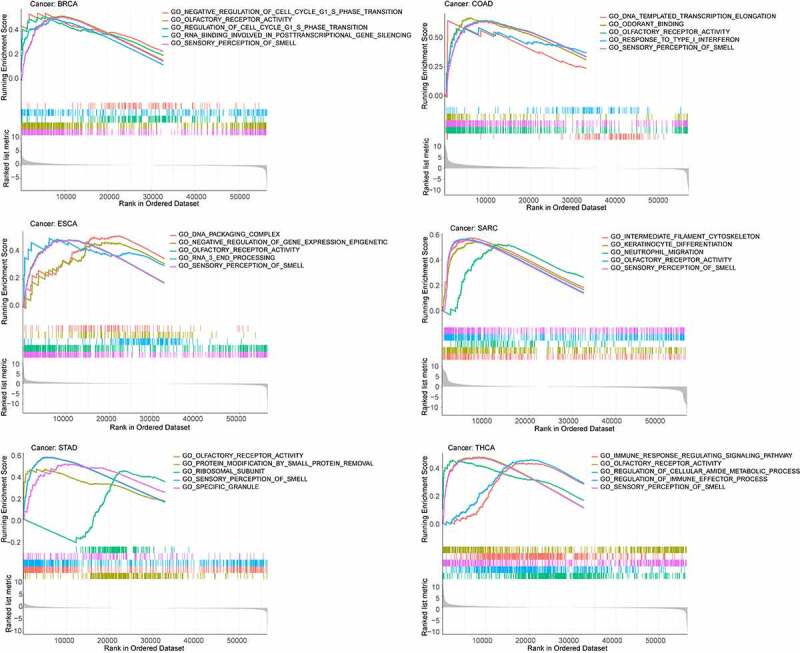
GO functional analysis of high *SPP1* expression.

To determine which signaling pathways associated with *SPP1* contribute to tumorigenesis, GSEA was performed, including functional analysis using gene ontology (GO) and the Kyoto Encyclopedia of Genes and Genomes (KEGG) (Table S9 and Table S10). We identified the top five signaling pathways in GO analysis and found that *SPP1* had a major impact on sensory perception of smell and olfactory receptor activity in most tumors. The higher the expression of *SPP1*, the more active these two pathways were in BRCA, COAD, ESCA, SRAC, STAD, and UCEC (Figure 7). The lower the expression of *SPP1*, the more active these two pathways were in BLCA, DLBC, OV, PRAD, READ, and THCA (Figure S9). The KEGG analysis showed that *SPP1* primary affects the olfactory transduction pathway in most tumors.

### *Knockdown* SPP1 *inhibit the proliferation and migration of tumor cells*

**Figure 8.**
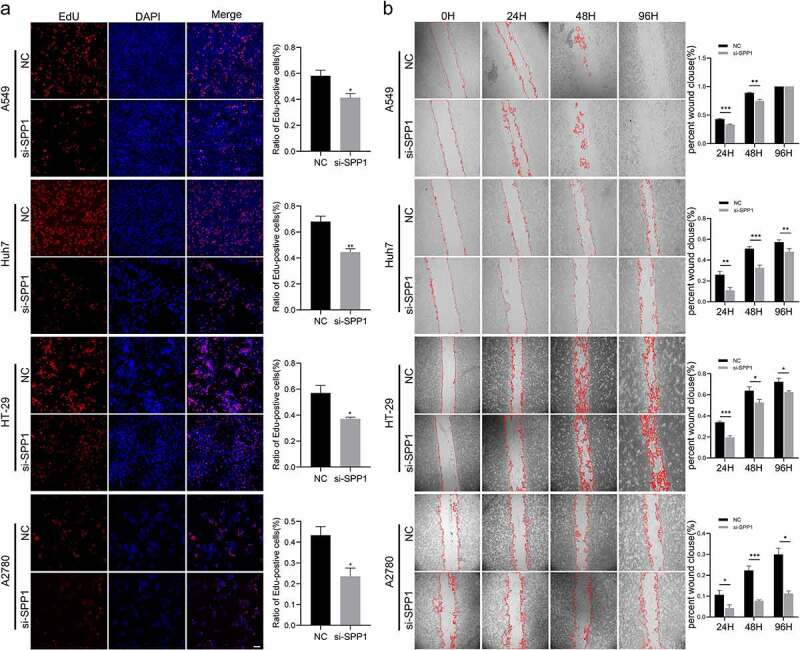
*SPP1* promote cell proliferation and migration in tumor cell lines. (a) Cell proliferation tested by EdU assay. (b) Cell migration detected by wound healing assay. (*P < 0.05, **P < 0.01, ***P < 0.001).

To further verify the oncogene features of *SPP1*, we evaluate the ability of *SPP1* in cell proliferation and migration. We used siRNA to knockdown *SPP1* expression in tumor cell lines. Following transfection, cell migration and proliferation were evaluated by EdU staining assay and wounding healing assay. The results showed that si-*SPP1* also decreased cell proliferation of these tumor cell lines (Figure 8(a)). We further investigated the effect of *SPP1* silencing on the cell migration. The results indicated that si-*SPP1* significantly decreased cell migration of A549, Huh-7, HT-29, and A2780 cell lines (Figure 8(b)).

## Discussion

Increasing evidence indicates that *SPP1* is an oncogene in many cancers [32–34]. Therefore, it is necessary to pay more attention to the role of *SPP1* in different aspects of tumor biology. In the present study, we analyzed the expression of *SPP1* in multiple tumors using the Oncomine and TCGA databases, revealing significantly differences of *SPP1* expression in various types of cancer. Compared with that in normal tissues, the *SPP1* mRNA level was significantly higher in most tumor tissues, including bladder, brain and central nervous system, cervical, colorectal, esophageal, gastric, head and neck, liver, lung, ovarian, pancreatic, and prostate cancer, and melanoma, while the expression trend of *SPP1* was inconsistent kidney cancer and sarcoma. In the TCGA database, *SPP1* expression was significant higher in most tumor tissues, but showed lower expression in KICH, PAAD, SKCM, and THYM, however, without statistical significance. The analysis results in the two databases were broadly similar. However, there were also some differences that might have been caused by the data collection and analysis methods. In addition, according to the Kaplan-Meier and COX survival analyses, we found that high expression of *SPP1* in most cancers is indicative of a poor prognosis, such as in CESC, ESCA, GBM, LGG, LIHC, LUAD, MESO, PAAD, PRAD, and STAD. Studies have also found that *SPP1* could promote the proliferation, migration, and invasion of malignant tumor cells and inhibit cell apoptosis, leading to a poor prognosis in certain tumors [35]. Increasing evidences indicate that *SPP1* is an important prognostic biomarker for many cancers [36,37].

The immune system normally recognizes and clears tumor cells in the tumor microenvironment. However, the growth of tumor cells also inhibits the immune system. When tumor cells are killed, the immune system is also suppressed. The biological function of *SPP1* has been observed in various immune cells and is involved in initiating immune responses [38]. Notably, in this study, we demonstrated that the *SPP1* expression was related to cancer immunity. *SPP1* expression was significantly associated with the degree of infiltration in macrophages (M0, M1, and M2), resting mast cells, B cells, and neutrophils in at least six types of cancer according to CIBERSORT analysis.

Previous studies suggested that *SPP1* promoted glioma progression by upregulating GBM-infiltrating neutrophils and macrophages, and was associated with infiltration of these cells within tumor specimens [39,40]. *SPP1* was also involved in neutrophil activation [41], and neutralization of *SPP1* attenuated neutrophil migration [42]. Currently, pan-cancer analysis employs the widely used ESTIMATE method to obtain immune and stromal scores in the tumor microenvironment, and to understand tumor prognosis [43,44]. In the present study, *SPP1* expression was observed to correlated positively with immune and stromal scores. Immune checkpoint inhibitors have revolutionized cancer treatment [45,46]. Therefore, we analyzed the expression relationship between *SPP1* and 47 common immune checkpoint genes. *SPP1* expression was observed to correlated positively with immune checkpoint gene expression in multiple types of cancer. Studies have demonstrated that *SPP1* could interact with CD44 in prostate cancer, GBM, and breast cancer [47,48]. Moreover, *SPP1* might trigger pro-inflammatory stimuli, such as that mediated by TNF [49]. Almost all members of the TNFSF have proinflammatory activity, which is partially activated by the nuclear factor kappa B (NF-κB). TNFRSFs are primarily transmembrane proteins involved in physiological processes, such as host defense, inflammation, apoptosis, and immune process [50]. As a checkpoint immunotherapies targets, the CTLA4 has shown remarkable success in the treatment of certain cancer types [51]. This study provided the basis for a deeper understanding of the potential mechanism of *SPP1* tumor immunity and its related cancer biomarkers. In recent years, immunotherapies have shown gradually increasing efficacy in treating tumors. Studies have demonstrated that immune checkpoint genes have a critical influence on immune cell infiltration and immunotherapy [52]. *SPP1* was likely correlated with immune cell infiltration and might contribute to the immunotherapy in hepatocellular carcinoma [53].This work found a strong relationship between *SPP1* and immune checkpoint genes, which provided a theoretical basis for combined molecular targeting immunotherapy in the future. We also found that *SPP1* was associated with immunotherapy response in BLCA, RCC, and SKCM. Therefore, *SPP1* might be a potential indicator in immunotherapy.

Genetic mutation is the major cause of tumorigenesis. Mutation and amplification were observed to be the main alteration frequency with mutation types of *SPP1*, especially in uterine cancer and melanoma. The TMB and MSI are emerging biomarkers that have predictive value in cancers [2,54,55]. The TMB has been widely studied as a predictive biomarker of the response to immune checkpoint inhibitors. To some extent, the TMB reflected the immunogenicity of the tumor, thus affecting the patient’s response to immune checkpoint inhibitors [56]. For example, the TMB determined the immune-related survival results of patients with breast cancer [57]. The high-quality and matched data from the TCGA contributed to a thorough investigation of the general prognostic impact of the TMB in patients newly diagnosed with cancer [56]. Previous studies showed that a high TMB was associated with better prognosis in STAD [58]; however a high TMB was associated with poor prognosis in non-small cell lung cancer [59]. Thus, the relationship between a high TMB and tumor prognosis might depend on the interaction between the tumor and the microenvironment. Recent studies showed that MSI was closely related to the occurrence and progression of many tumors. Microsatellite mutation can cause normal cells to transform into malignant cells. MSI was reported to be increased significantly in a variety of cancer tissues [60,61]. Therefore, MSI has become an important diagnostic index to screen malignant tumors. Lu et al have already found that olfactory transduction pathway could affect apoptosis of lung cancer cells and might be new hallmark of lung cancer [62]. The olfactory transduction is the main signaling pathway enriched from the unique subset of genes identified in esophageal squamous cell carcinoma [63]. Our study also found that *SPP1* had a major impact on olfactory transduction. However, the underlying mechanisms requires further investigation.

## Conclusions

In summary, our findings suggested that *SPP1* could regulate tumorigenesis, tumor progression, and prognosis. The expression of *SPP1* was significantly different in diverse human cancers. Therefore, *SPP1* expression could be a valuable prognostic biomarker, because *SPP1* upregulation resulted in a significant decrease in patient survival in certain cancer types. In addition, we revealed that the *SPP1* expression was related to cancer immunity. Immunotherapy did not affect the immune/stromal score in LGG, and

affected immune/stromal score in COAD and OV. Therefore, *SPP1* may serve as an attractive target in cancer mechanistic research and in treatment.

## Supplementary Material

Supplemental Material

## Data Availability

Publicly available datasets were analyzed in this article. These data can be found here: Oncomine database (http://www.oncomine.org/resource/login.html), UCSC Xena website (http://xena.ucsc.edu/), and the GSEA website (http://www.gsea-msigdb.org/gsea/index.jsp).
