# Supplementary material for: Secreted phosphoprotein 1 as a potential prognostic and immunotherapy biomarker in multiple human cancers
Source: Bioengineered. 2022 Jan 22;13(2):3221–39. doi: 10.1080/21655979.2021.2020391 (PMC8973783; doi:10.1080/21655979.2021.2020391)
Supplement: Supplemental Material [file KBIE_A_2020391_SM0415.zip › supplementary/Supplementary material.DOCX]

*Supplementary Material*

SPP1 as a potential prognostic and immunotherapy biomarker in human cancer

Ping Zeng^1, †^, Xujun Zhang^1, †^, Tianxin Xiang^2^, Zongxin Ling^1^, Chenhong Lin^1^, Hongyan Diao^1*^

^1^ State Key Laboratory for Diagnosis and Treatment of Infectious Diseases, National Clinical Research Center for Infectious Diseases, Collaborative Innovation Center for Diagnosis and Treatment of Infectious Diseases, The First Affiliated Hospital, College of Medicine, Zhejiang University, Hangzhou, China.

^2^ Department of Hospital Infection Control, The First Affiliated Hospital of Nanchang University, Nanchang, China.

*** Correspondence:**Hongyan Diao
[diaohy@zju.edu.cn](mailto:diaohy@zju.edu.cn)

† These authors have contributed equally to this work and share authorship.


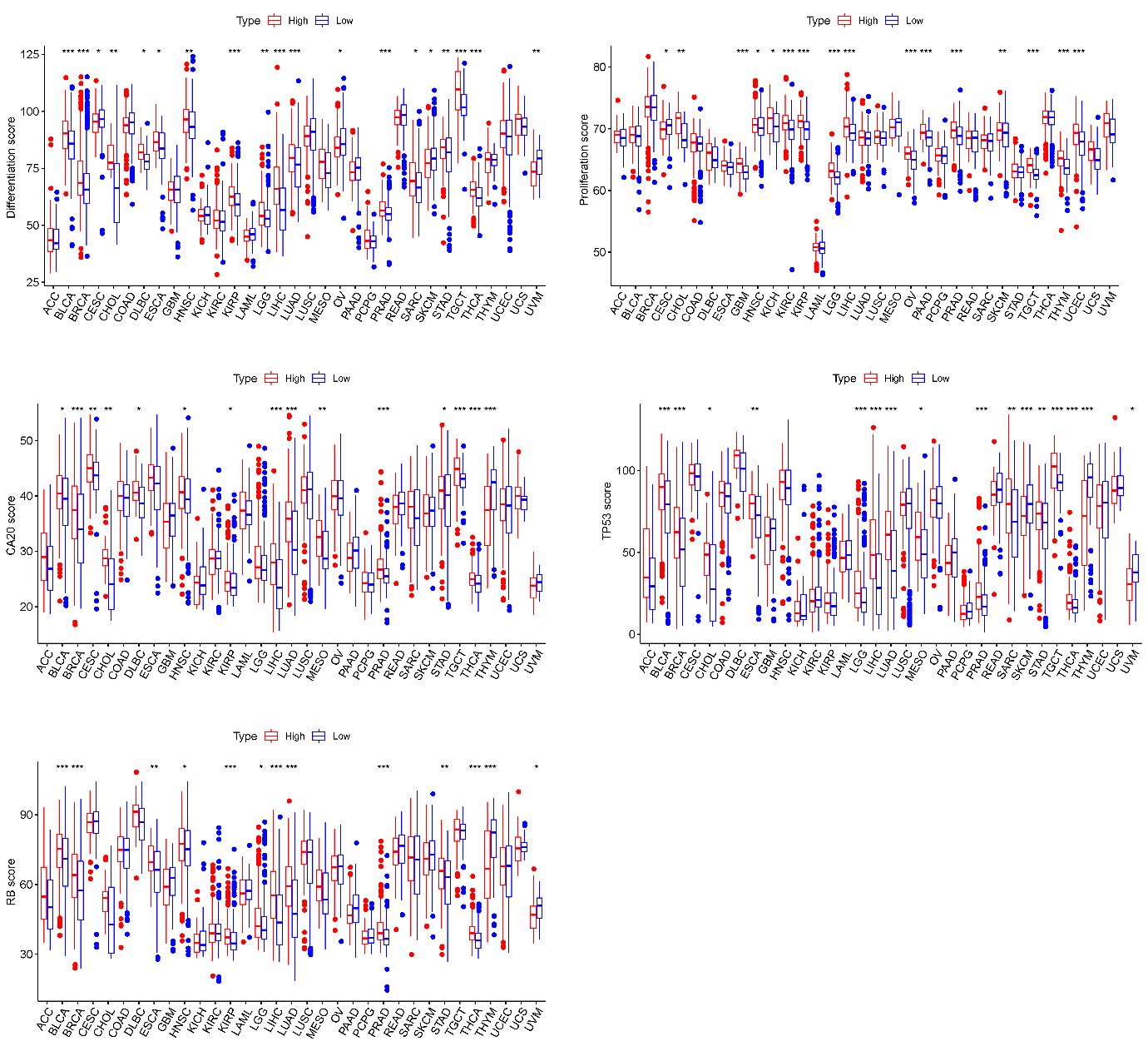


**Supplementary Figure 1.** Differentiation, Proliferation, CA20, TP53, and RB signature scores for TCGA samples.


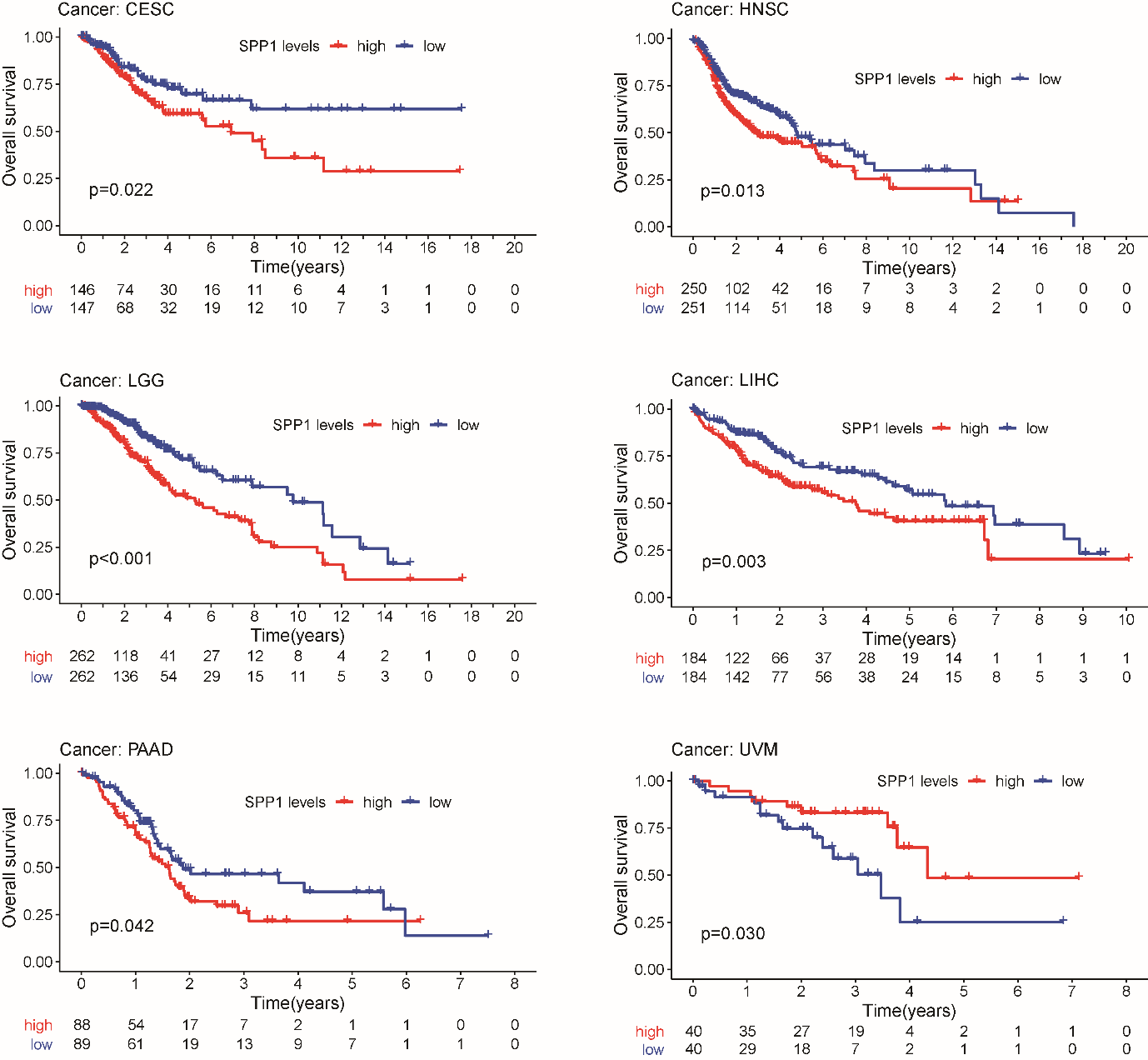


**Supplementary Figure 2.** Analysis of the association between overall survival and *SPP1* expression in different cancer types.
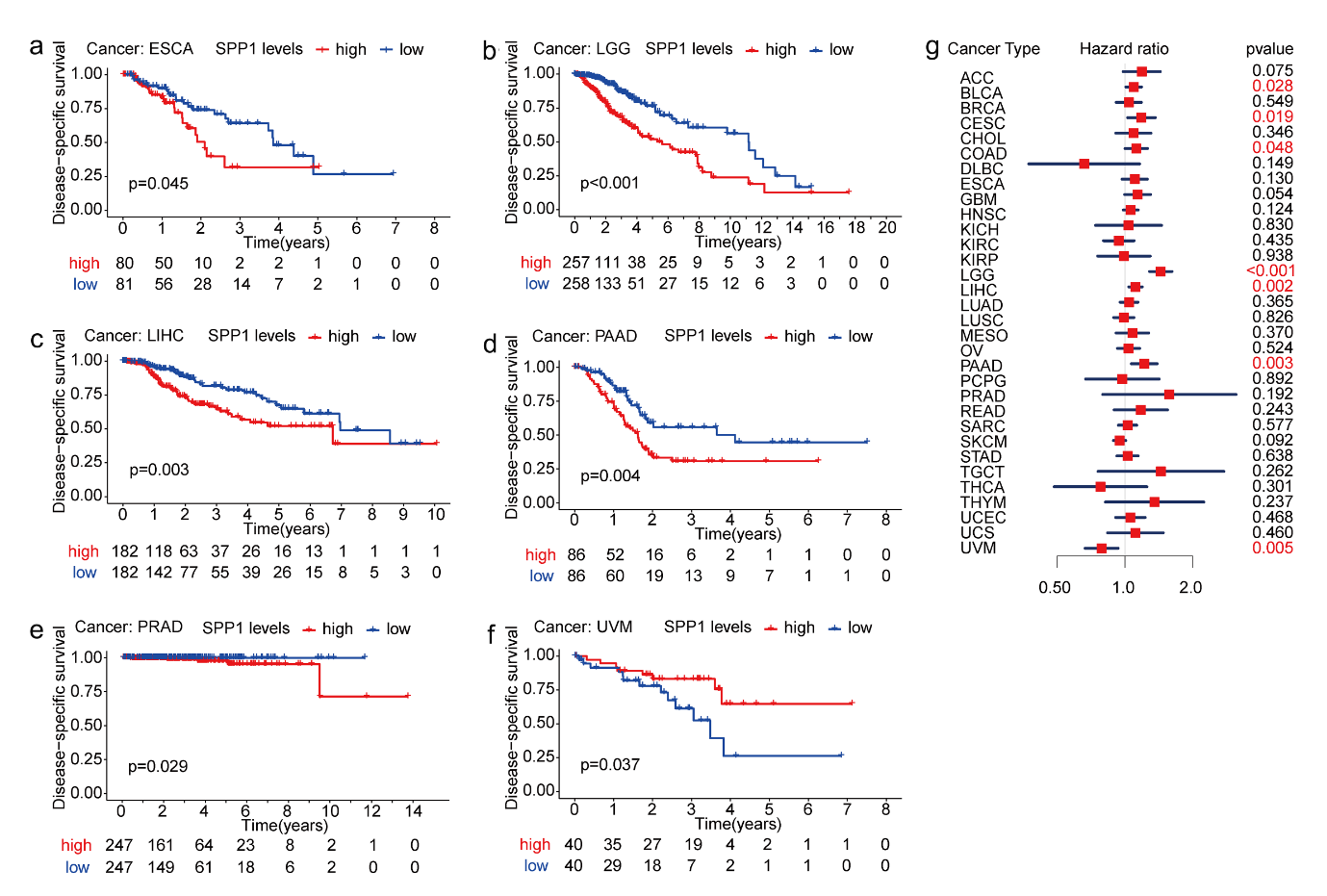


**Supplementary Figure 3.** Disease-specific survival analysis of the *SPP1* expression in different cancer types.


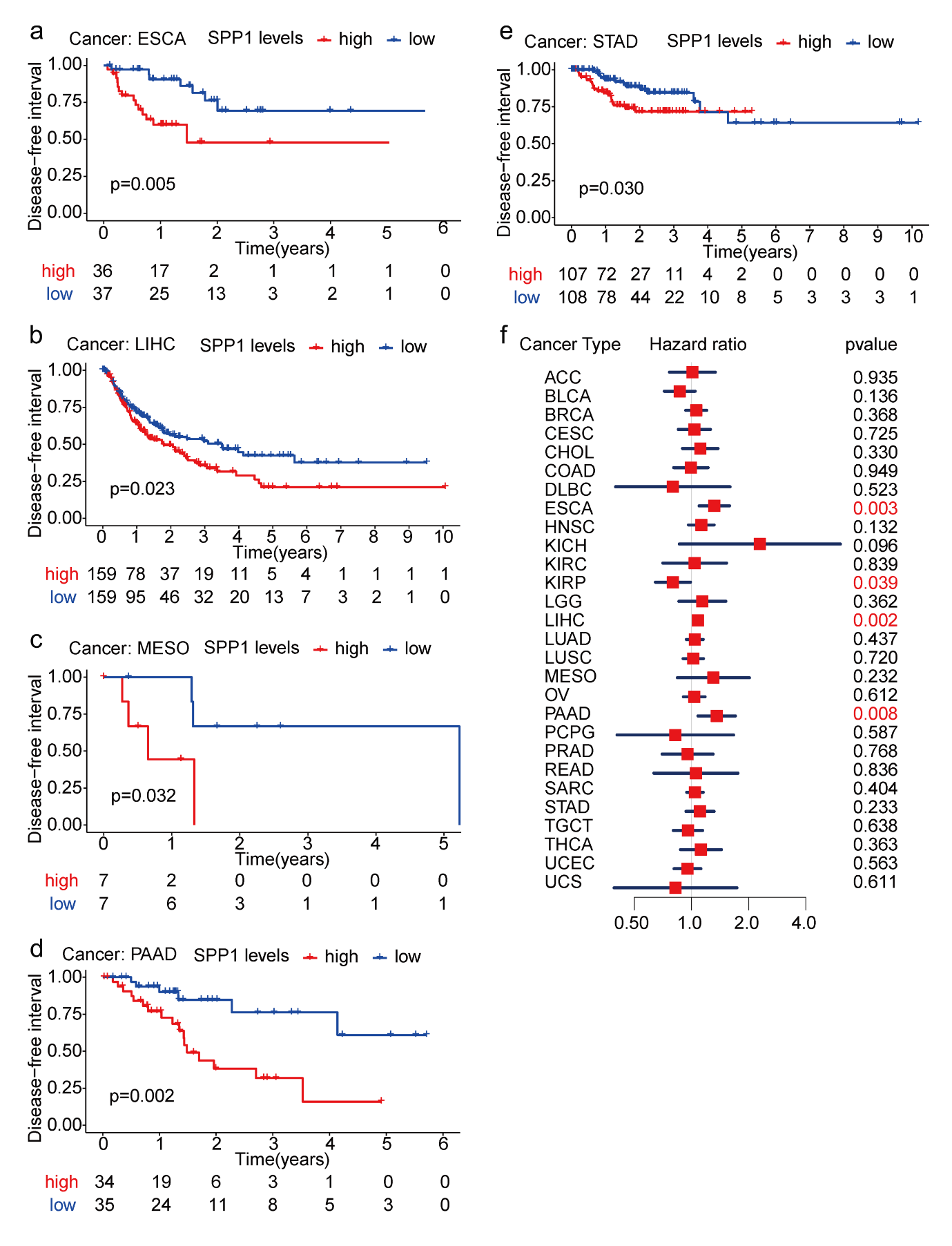


**Supplementary Figure 4.** Disease-free interval analysis of the *SPP1* expression in different cancer types.


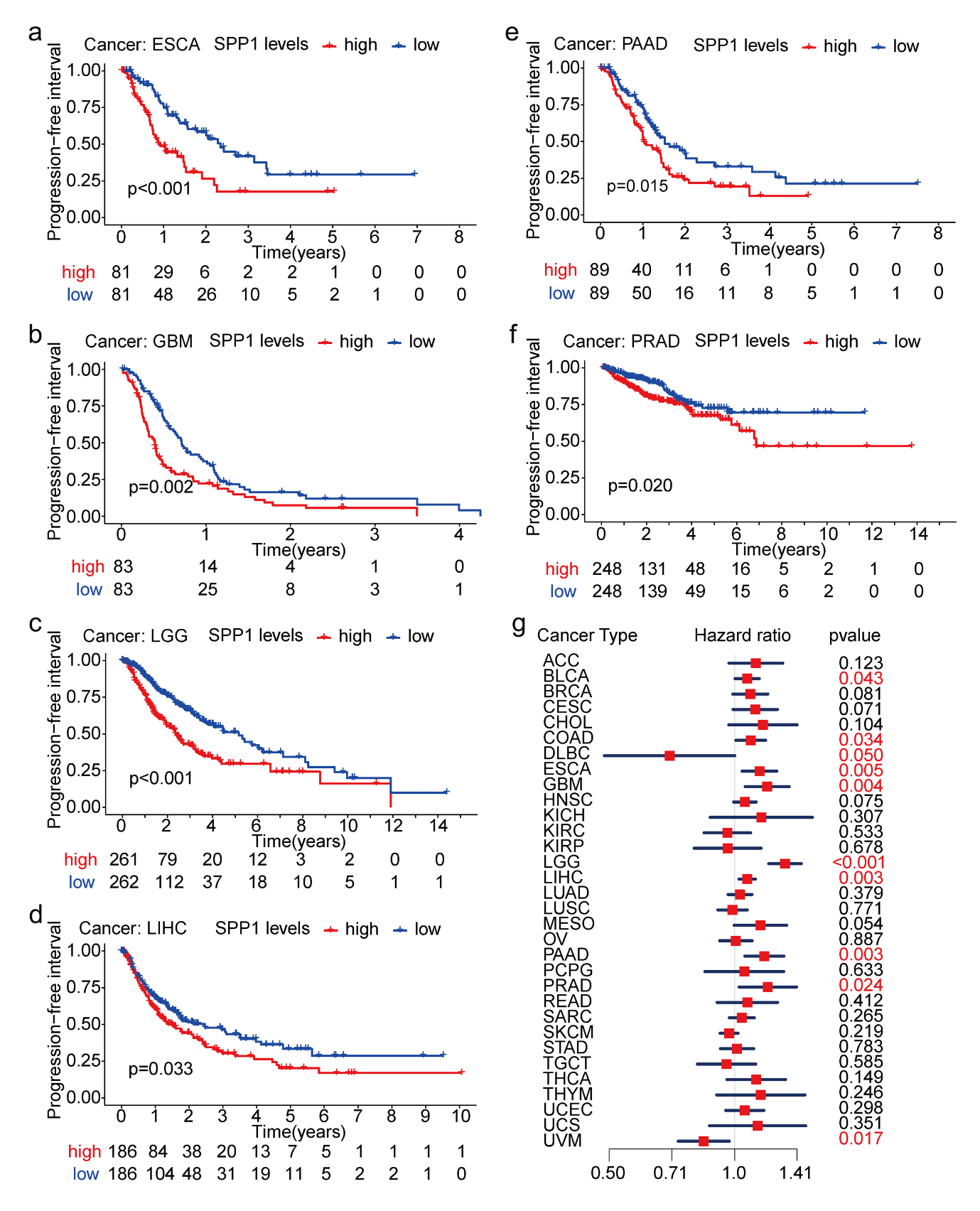


**Supplementary Figure 5.** Progression-free survival analysis of the *SPP1* expression in different cancer types.


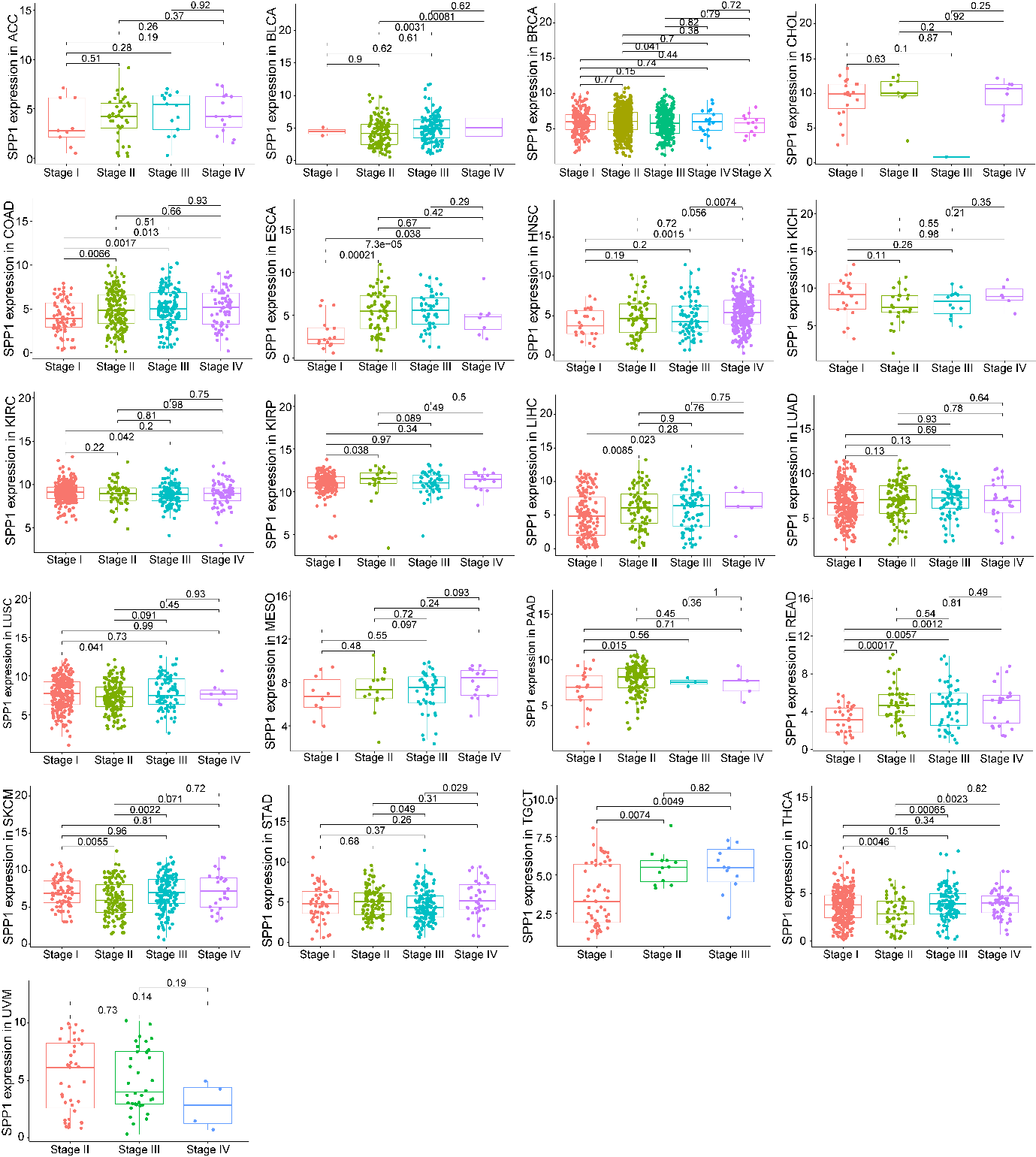


**Supplementary Figure 6.** The association of *SPP1* expression with histological grade for 16 cancer types.


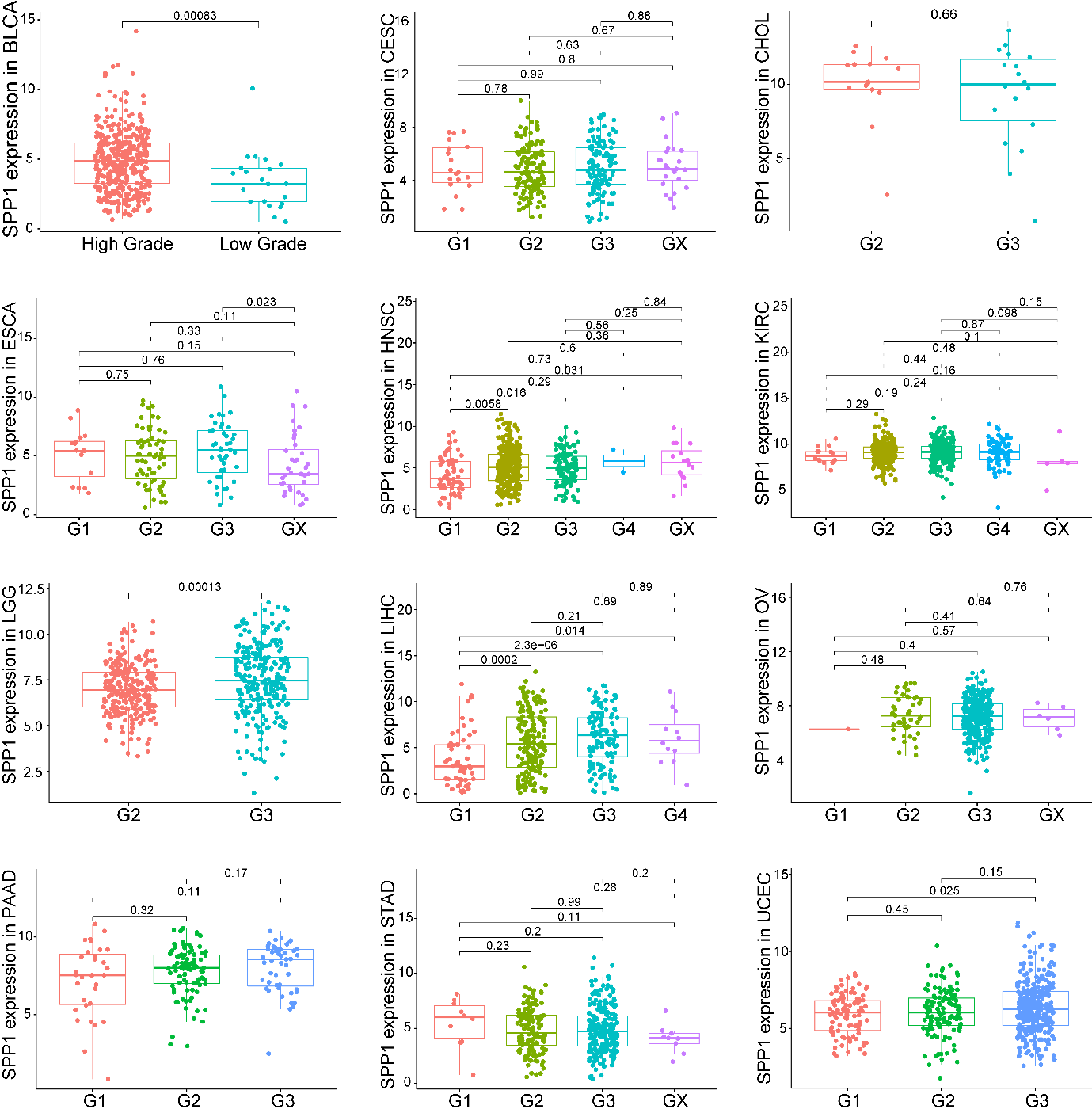


**Supplementary Figure 7.** The association of *SPP1* expression with pathological stages for 21 cancer types.


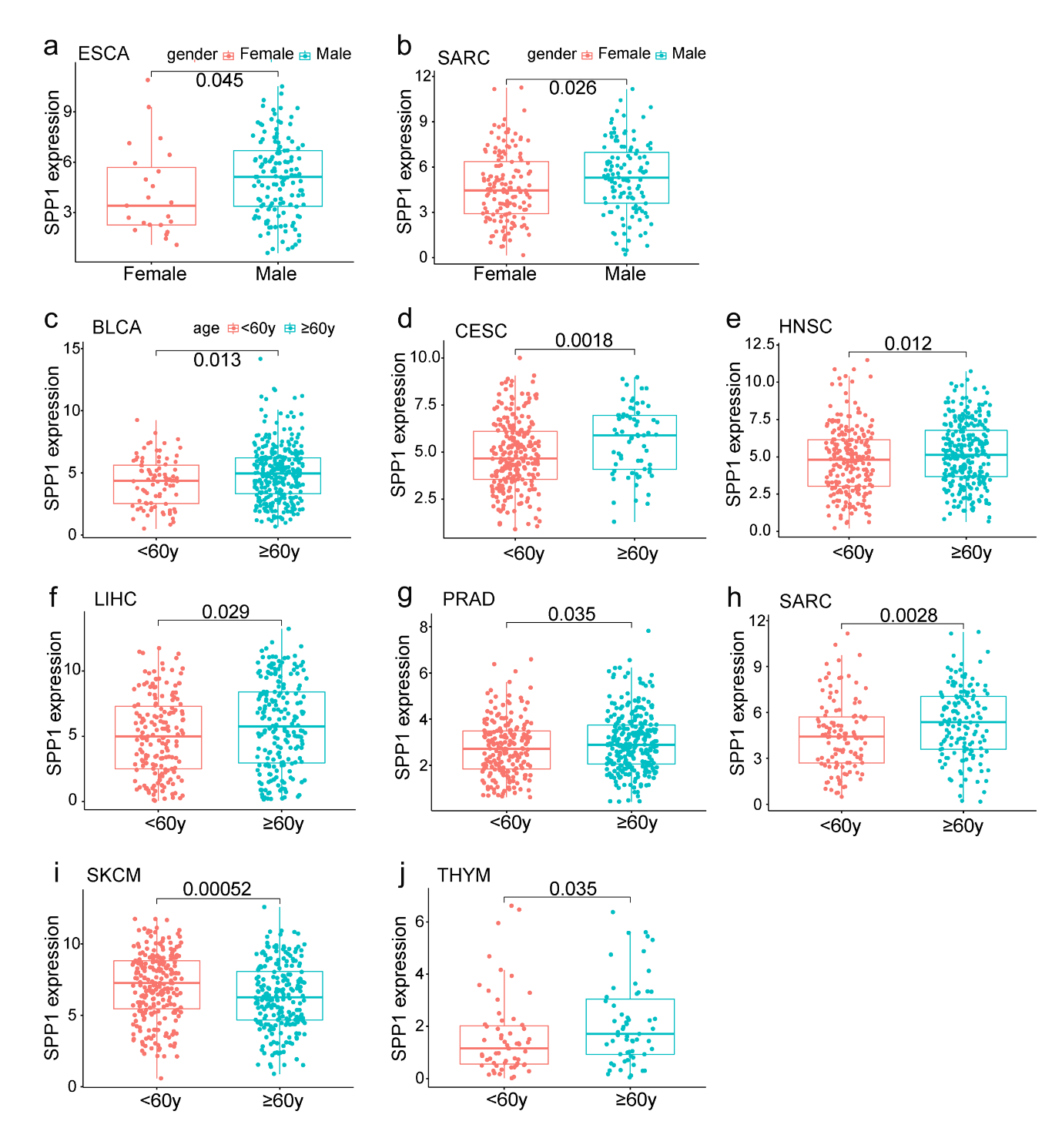


**Supplementary Figure 8.** Correlation of *SPP1* expression with sex and age in different cancer types. **(a, b** The association of *SPP1* expression with sex differences in ESCA and SARC. **(c-j)** The association of *SPP1* expression with age differences in BLCA, CESC, HNSC, LIHC, PRAD, SARC, SKCM, and THYM. Only P<0.05 is shown.

**
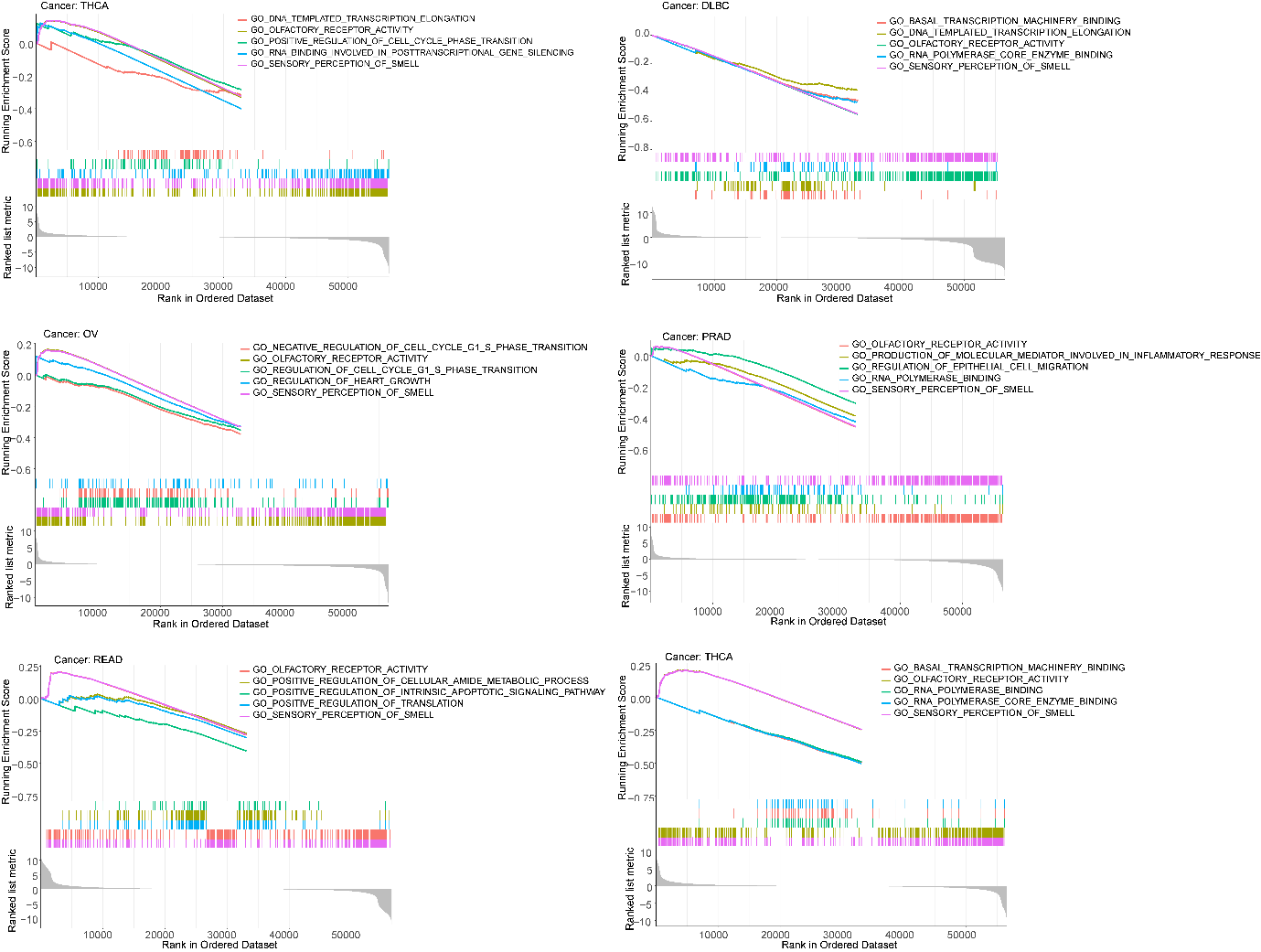
**

**Supplementary Figure 9.** GO functional analysis of low *SPP1* expression.

**Supplementary Table 1**. *SPP1* expression in cancers versus normal tissue in oncomine database. (P-value:0.001, Fold change:2, Gene rank: top 10%, Data Type: all)

| Cancer | Cancer Type | P-value | Fold change | Rank (%) | Sample | Reference (PMID) |
| --- | --- | --- | --- | --- | --- | --- |
| Bladder Cancer | Infiltration Bladder Urothelial Carcinoma | 1.19e-14 | 6.229 | 2% | 129 | 16432078 |
|  | Infiltration Bladder Urothelial Carcinoma | 1.08e-6 | 9.055 | 2% | 27 | 15173019 |
|  | Infiltration Bladder Urothelial Carcinoma | 6.22e-6 | 2.583 | 2% | 130 | 20421545 |
| Brain and CNS Cancer | Glioblastoma | 1.10e-7 | 5.740 | 1% | 15 | TCGA |
|  | Glioblastoma | 1.03e-6 | 2.346 | 4% | 31 | 16204036 |
|  | Glioblastoma | 2.21e-4 | 129.293 | 5% | 25 | 16697959 |
| Breast Cancer | Invasion Ductal Breast Carcinoma Stroma | 2.87e-5 | 16.337 | 1% | 22 | 17914389 |
|  | Ductal Breast Carcinoma in Situ | 7.62e-5 | 7.786 | 2% | 154 | 22522925 |
|  | Invasion Breast Carcinoma | 7.36e-6 | 4.068 | 5% | 163 | 22522925 |
|  | Mucinous Breast Carcinoma | 4.48e-13 | 4.116 | 6% | 190 | 22522925 |
|  | Tubular Breast Carcinoma | 4.30e-19 | 4.813 | 6% | 211 | 22522925 |
|  | Invasion Ductal and Invasion Lobular Breast Carcinoma | 5.72e-19 | 3.676 | 7% | 234 | 22522925 |
|  | Invasion Ductal Breast Carcinoma | 1.69e-48 | 3.980 | 9% | 1700 | 22522925 |
|  | Invasion Breast Carcinoma Type: Basal-Like Subtype of Invasion Breast Carcinoma | 1.17e-4 | 2.601 | 4% | 43 | 15897907 |
|  | Invasion Breast Carcinoma | 7.15e-15 | 4.740 | 6% | 137 | TCGA |
| Cervical Cancer | Cervical Squamous Cell Carcinoma | 1.93e-15 | 18.837 | 1% | 56 | 18506748 |
|  | High Grade Cervical Squamous Intraepithelial Neoplasia Epithelia | 2.37e-5 | 2.442 | 1% | 17 | 17974957 |
|  | Cervical Squamous Cell Carcinoma Epithelia | 3.43e-7 | 5.294 | 2% | 31 | 17974957 |
|  | Cervical Cancer | 2.55e-8 | 11.438 | 4% | 42 | 17510386 |
| Colorectal Cancer | Colon Mucinous Adenocarcinoma | 7.35e-11 | 35.194 | 1% | 18 | 17615082 |
|  | Cecum Adenocarcinoma | 5.54e-10 | 18.063 | 1% | 22 | 17615082 |
|  | Colon Adenocarcinoma | 1.50e-11 | 13.170 | 2% | 46 | 17615082 |
|  | Colon Mucinous Adenocarcinoma | 1.20e-14 | 35.002 | 1% | 44 | TCGA |
|  | Colon Adenocarcinoma | 4.11e-20 | 5.250 | 3% | 123 | TCGA |
|  | Rectal Adenocarcinoma | 1.66e-12 | 3.688 | 8% | 82 | TCGA |
|  | Colon Adenocarcinoma | 1.79e-04 | 4.988 | 4% | 36 | 11306497 |
|  | Colorectal Carcinoma | 9.44e-12 | 14.795 | 2% | 60 | 20957034 |
|  | Colon Carcinoma | 1.56e-9 | 44.932 | 2% | 15 | 20957034 |
|  | Colon Carcinoma Epithelia | 6.89e-6 | 31.724 | 9% | 15 | 20957034 |
|  | Colorectal Carcinoma | 1.91e-12 | 4.127 | 3% | 82 | 20143136 |
| Esophageal Cancer | Esophageal Squamous Cell Carcinoma | 1.99e-22 | 9.154 | 1% | 106 | 21385931 |
|  | Esophageal Squamous Cell Carcinoma | 3.89e-9 | 24.630 | 1% | 34 | 20955586 |
|  | Esophageal Adenocarcinoma | 4.58e-4 | 5.257 | 3% | 16 | 15833844 |
|  | Esophageal Adenocarcinoma | 3.28e-11 | 3.515 | 5% | 103 | 21152079 |
| Gastric Cancer | Gastric Cancer | 2.52e-10 | 4.042 | 1% | 160 | 20965966 |
|  | Diffuse Gastric Adenocarcinoma | 5.07e-10 | 5.999 | 1% | 50 | 21447720 |
|  | Gastric Intestinal Type Adenocarcinoma | 1.71e-4 | 3.587 | 4% | 39 | 21447720 |
|  | Gastric Intestinal Type Adenocarcinoma | 2.58e-13 | 15.519 | 1% | 57 | 19081245 |
|  | Gastric Cancer | 8.32e-5 | 7.611 | 2% | 27 | 21132402 |
| Head and Neck Cancer | Head and Neck Squamous Cell Carcinoma | 13.1e-20 | 43.614 | 1% | 54 | 14729608 |
|  | Oral Cavity Squamous Cell Carcinoma | 8.04e-24 | 11.215 | 1% | 79 | 21853135 |
|  | Head and Neck Squamous Cell Carcinoma | 3.81e-6 | 25.599 | 1% | 38 | 21853135 |
|  | Tongue Squamous Cell Carcinoma | 2.16e-6 | 2.576 | 1% | 38 | 18254958 |
|  | Tongue Squamous Cell Carcinoma | 2.52e-9 | 15.528 | 4% | 57 | 19138406 |
|  | Salivary Gland Adenoid Cystic Carcinoma | 1.78e-5 | 83.241 | 4% | 22 | 12368205 |
|  | Oral Cavity Squamous Cell Carcinoma Epithelia | 1.79e-5 | 4.598 | 3% | 20 | 15381369 |
|  | Tongue Squamous Cell Carcinoma | 7.68e-8 | 5.325 | 7% | 59 | 15833835 |
|  | Floor of the Mouth Carcinoma | 8.17e-5 | 10.398 | 5% | 27 | 17510386 |
| Kidney Cancer | Papillary Renal Cell Carcinoma | 1.11e-8 | 2.740 | 6% | 34 | 16115910 |
|  | Clear Cell Sarcoma of the Kidney | 3.07e-5 | -3.934 | 3% | 17 | 16299227 |
|  | Clear Cell Renal Cell Carcinoma | 6.91e-4 | -2.319 | 6% | 18 | 14641932 |
| Liver Cancer | Hepatocellular Carcinoma | 1.66e-10 | 7.505 | 2% | 57 | 19098997 |
|  | Cirrhosis | 5.68e-9 | 5.397 | 9% | 67 | 19098997 |
|  | Hepatocellular Carcinoma | 3.46e-6 | 3.002 | 4% | 45 | 17393520 |
|  | Hepatocellular Carcinoma | 1.53e-9 | 4.016 | 6% | 179 | 12058060 |
|  | Hepatocellular Carcinoma | 5.24e-6 | 8.999 | 7% | 43 | 21159642 |
| Lung Cancer | Lung Adenocarcinoma | 6.73e-38 | 20.616 | 1% | 107 | 18297132 |
|  | Lung Adenocarcinoma | 8.84e-23 | 16.376 | 1% | 110 | 20421987 |
|  | Squamous Cell Lung Carcinoma | 1.72e-23 | 21.100 | 1% | 92 | 20421987 |
|  | Large Cell Lung Carcinoma | 4.79e-7 | 9.093 | 4% | 84 | 20421987 |
|  | Squamous Cell Lung Carcinoma | 1.02e-18 | 20.369 | 1% | 62 | 20421987 |
|  | Lung Adenocarcinoma | 3.98e-16 | 14.731 | 1% | 57 | 17540040 |
|  | Lung Adenocarcinoma | 8.56e-13 | 15.011 | 1% | 39 | 16314486 |
|  | Lung Adenocarcinoma | 1.55e-5 | 23.783 | 1% | 149 | 11707567 |
|  | Squamous Cell Lung Carcinoma | 5.67e-7 | 60.245 | 2% | 38 | 11707567 |
|  | Lung Adenocarcinoma | 6.79e-24 | 10.445 | 1% | 116 | 22613842 |
|  | Squamous Cell Lung Carcinoma | 1.49e-4 | 26.391 | 2% | 10 | 16188928 |
|  | Squamous Cell Lung Carcinoma | 8.91e-5 | 14.452 | 3% | 19 | 11707590 |
|  | Lung Adenocarcinoma | 4.18e-4 | 9.467 | 5% | 46 | 11707590 |
|  | Lung Adenocarcinoma | 2.43e-4 | 6.877 | 9% | 96 | 12118244 |
|  | Lung Adenocarcinoma | 2.07e-9 | 15.546 | 9% | 246 | 22080568 |
| Lymphoma | Cutaneous Follicular Lymphoma | 1.41e-4 | 2.905 | 1% | 14 | 12713594 |
|  | Primary Effusion Lymphoma | 6.97e-8 | 137.979 | 2% | 34 | 15778709 |
|  | Centroblastic Lymphoma | 2.99e-9 | 12.803 | 6% | 53 | 15778709 |
|  | Diffuse Large B-Cell Lymphoma | 5.55e-18 | 12.096 | 3% | 64 | 19412164 |
|  | Germinal Center B-Cell-Like Diffuse Large B-Cell Lymphoma | 3.92e-4 | 5.406 | 10% | 29 | 19412164 |
|  | Unspecified Peripheral T-Cell Lymphoma | 1.31e-9 | 8.739 | 7% | 48 | 17304354 |
| Melanoma | Cutaneous Melanoma | 6.43e-8 | 13.322 | 3% | 52 | 16243793 |
|  | Melanoma | 4.19e-4 | 37.604 | 5% | 9 | 15833814 |
| Ovarian Cancer | Ovarian Serous Adenocarcinoma | 3.12e-5 | 25.623 | 1% | 10 | 14760385 |
|  | Ovarian Serous Adenocarcinoma | 6.61e-7 | 31.013 | 4% | 53 | 19486012 |
| Pancreatic Cancer | Pancreatic Ductal Adenocarcinoma | 1.31e-4 | 2.700 | 2% | 14 | 16103885 |
|  | Pancreatic Ductal Adenocarcinoma | 4.26e-11 | 6.619 | 3% | 78 | 19260470 |
| Prostate Cancer | Prostate Adenocarcinoma | 1.23e-4 | 2.185 | 4% | 35 | 12873976 |
|  | Prostate Carcinoma | 1.95e-5 | 3.004 | 5% | 87 | 22722839 |
| Sarcoma | Pleomorphic Liposarcoma | 2.88e-4 | 33.671 | 3% | 18 | 15994966 |
|  | Malignant Fibrous Histiocytoma | 9.15e-4 | 9.821 | 10% | 24 | 15994966 |
|  | Clear Cell Sarcoma of the Kidney | 3.07e-5 | -3.934 | 3% | 17 | 16299227 |
| Other Cancer | Adrenal Cortex Carcinoma | 2.55e-8 | 2.044 | 2% | 39 | 19147773 |
|  | Pleural Malignant Mesothelioma | 1.46e-4 | 11.128 | 6% | 49 | 15920167 |
|  | Vulvar Intraepithelial Neoplasia | 4.39e-4 | 6.552 | 6% | 19 | 17471573 |
|  | Malignant Fibrous Histiocytoma | 9.15e-4 | 9.821 | 10% | 24 | 15994966 |
|  | Teratoma, NOS | 1.69e-6 | 14.032 | 8% | 20 | 16424014 |
|  | Yolk Sac Tumor, NOS | 8.85e-5 | 7.717 | 9% | 15 | 16424014 |

**Supplementary Table 2**. The clinicopathological features of cancer patients

| Variables | COAD | LIHC | STAD | BRCA |
| --- | --- | --- | --- | --- |
| Age |  |  |  |  |
| ＞60 | 9 | 12 | 11 | 8 |
| ≤60 | 11 | 8 | 9 | 12 |
| Gender |  |  |  |  |
| Male | 14 | 17 | 15 | 0 |
| Female | 6 | 7 | 5 | 20 |
| TNM stage |  |  |  |  |
| Ⅰ+Ⅱ | 12 | 11 | 14 | 11 |
| Ⅲ+Ⅳ | 8 | 9 | 6 | 9 |
| Tumor differentiation |  |  |  |  |
| Well+moderate | 11 | 13 | 11 | 13 |
| Poor | 9 | 7 | 9 | 7 |
|  | | | | |

**Supplementary Table 3**. The SPP1 expression level between tumor and para-tumor tissues

| Samples | n=20 | COAD | | p**^a^** | LIHC | | p**^a^** | STAD | | p**^a^** | BRCA | | P**^a^** |
| --- | --- | --- | --- | --- | --- | --- | --- | --- | --- | --- | --- | --- | --- |
|  |  | Low | High |  | Low | High |  | Low | High |  | Low | High |  |
| Tumor | 20 | 6 | 14 | 0.027 | 8 | 12 | 0.028 | 7 | 13 | 0.011 | 5 | 15 | 0.004 |
| Para-tumor | 20 | 13 | 7 |  | 14 | 6 |  | 15 | 5 |  | 14 | 6 |  |
| a: **χ**^2^ test. | | | | | | | | | | | | | |

**Supplementary Table 4.** The association of *SPP1* expression with gender and age for 33 cancer types.

| Cancer Type | Gender | | | Age | | |
| --- | --- | --- | --- | --- | --- | --- |
|  | **Female(n)** | **Male(n)** | **P value** | **<60y(n)** | **≥60y(n)** | **P value** |
| ACC | 60 | 32 | 0.64 | 68 | 24 | 0.26 |
| BLCA | 119 | 317 | 0.14 | 95 | 341 | **0.013** |
| BRCA | 1223 | 13 | 0.81 | 667 | 568 | 0.44 |
| CESC | 312 | 0 | - | 245 | 67 | **0.0018** |
| CHOL | 23 | 22 | 0.4 | 13 | 32 | 0.52 |
| COAD | 261 | 284 | 0.19 | 145 | 400 | 0.64 |
| DLBC | 26 | 22 | 0.6 | 26 | 22 | 0.36 |
| ESCA | 33 | 171 | **0.045** | 89 | 115 | 0.8 |
| GBM | 234 | 368 | 0.54 | 314 | 288 | 0.76 |
| HNSC | 164 | 440 | 0.93 | 263 | 340 | **0.012** |
| KICH | 39 | 52 | 0.61 | 64 | 27 | 0.38 |
| KIRC | 237 | 617 | 0.67 | 431 | 513 | 0.39 |
| KIRP | 95 | 257 | 0.082 | 144 | 205 | 0.69 |
| LAML | 91 | 109 | 0.16 | 108 | 92 | 0.97 |
| LGG | 238 | 291 | 0.49 | 459 | 70 | 0.26 |
| LIHC | 146 | 292 | 0.14 | 191 | 246 | **0.029** |
| LUAD | 349 | 292 | 0.9 | 168 | 454 | 0.56 |
| LUSC | 165 | 458 | 0.084 | 112 | 502 | 0.66 |
| MESO | 16 | 71 | 0.63 | 27 | 60 | 0.82 |
| OV | 604 | 0 | - | 321 | 283 | 0.27 |
| PAAD | 87 | 109 | 0.94 | 62 | 134 | 0.86 |
| PCPG | 103 | 84 | 0.052 | 145 | 42 | 0.67 |
| PRAD | 0 | 566 | - | 229 | 337 | **0.035** |
| READ | 86 | 97 | 0.66 | 60 | 123 | 0.74 |
| SARC | 149 | 122 | **0.026** | 120 | 151 | **0.0028** |
| SKCM | 183 | 296 | 0.47 | 248 | 223 | **0.00052** |
| STAD | 190 | 321 | 0.55 | 146 | 360 | 0.24 |
| TGCT | 0 | 139 | - | 137 | 2 | 0.54 |
| THCA | 423 | 157 | 0.12 | 443 | 137 | 0.68 |
| THYM | 62 | 64 | 0.34 | 62 | 63 | **0.035** |
| UCEC | 583 | 0 | - | 195 | 385 | 0.65 |
| UCS | 57 | 0 | - | 6 | 51 | 0.93 |
| UVM | 35 | 45 | 0.56 | 36 | 44 | 0.7 |

**Supplementary Table 5.** Correlation analysis of *SPP1* expression with TMB and MSI for 33 cancer types.

| Cancer Type | TMB | | MSI | |
| --- | --- | --- | --- | --- |
|  | cor | pValue | cor | pValue |
| ACC | 0.263482 | 0.018967 | 0.142431 | 0.210507 |
| BLCA | 0.08316 | 0.093443 | -0.03536 | 0.476277 |
| BRCA | 0.045208 | 0.158599 | -0.01541 | 0.621069 |
| CESC | 0.182628 | 0.001928 | 0.108837 | 0.05887 |
| CHOL | 0.211259 | 0.216149 | 0.054311 | 0.752393 |
| COAD | 0.193104 | 0.00011 | 0.288664 | 1.22E-09 |
| DLBC | 0.405168 | 0.013431 | 0.09141 | 0.536635 |
| ESCA | -0.16148 | 0.041349 | 0.030629 | 0.700623 |
| GBM | -0.12777 | 0.12173 | -0.18947 | 0.019803 |
| HNSC | 0.073128 | 0.105211 | -0.05347 | 0.234569 |
| KICH | -0.00836 | 0.947324 | -0.14604 | 0.245732 |
| KIRC | 0.15283 | 0.005261 | 0.065978 | 0.228439 |
| KIRP | 0.086133 | 0.152054 | 0.008983 | 0.879984 |
| LAML | 0.16353 | 0.200325 | 0.023271 | 0.850592 |
| LGG | 0.102873 | 0.021281 | -0.0471 | 0.289352 |
| LIHC | 0.000628 | 0.99054 | 0.072546 | 0.164324 |
| LUAD | 0.039525 | 0.376381 | -0.14428 | 0.001073 |
| LUSC | -0.07522 | 0.096973 | -0.21147 | 2.17E-06 |
| MESO | 0.06491 | 0.569803 | -0.16279 | 0.143944 |
| OV | 0.124389 | 0.040364 | -0.1258 | 0.038132 |
| PAAD | -0.11676 | 0.153366 | -0.16119 | 0.033092 |
| PCPG | -0.06301 | 0.404716 | 0.017464 | 0.817019 |
| PRAD | 0.203449 | 6.73E-06 | 0.032165 | 0.475228 |
| READ | -0.00953 | 0.913671 | 0.125704 | 0.122803 |
| SARC | 0.286246 | 8.26E-06 | 0.202047 | 0.001233 |
| SKCM | -0.02208 | 0.634856 | 0.013746 | 0.766787 |
| STAD | 0.183639 | 0.000399 | 0.060231 | 0.245249 |
| TGCT | 0.025773 | 0.758301 | 0.159715 | 0.050904 |
| THCA | -0.04773 | 0.29566 | -0.01555 | 0.731051 |
| THYM | 0.30189 | 0.00094 | -0.06897 | 0.458043 |
| UCEC | 0.055538 | 0.203917 | 0.036698 | 0.395596 |
| UCS | 0.152844 | 0.260756 | -0.11754 | 0.388291 |
| UVM | -0.07442 | 0.511757 | -0.11676 | 0.302344 |

**Supplementary Table 6.** Correlation analysis of *SPP1* expression with immune and stromal score for 23 cancer types (Data for presentation only P < 0.001).

| Cancer types | Immune Score | | Stromal Score | |
| --- | --- | --- | --- | --- |
|  | R | P value | R | P value |
| BLCA | 0.42 | <2.2E-16 | 0.48 | <2.2E-16 |
| BRCA | 0.15 | 1.30E-06 | 0.23 | 3.50E-14 |
| CESC |  |  | 0.26 | 5.50E-06 |
| COAD | 0.5 | <2.2E-16 | **0.69** | <2.2E-16 |
| ESCA |  |  | 0.39 | 3.10E-07 |
| GBM | 0.6 | <2.2E-16 | 0.52 | <2.2E-16 |
| HNSC |  |  | 0.3 | 1.60E-11 |
| LGG | **0.69** | <2.2E-16 | **0.63** | <2.2E-16 |
| LIHC | 0.26 | 3.10E-07 |  |  |
| LUAD | 0.17 | 5.80E-05 | 0.32 | 7.60E-14 |
| LUSC | 0.16 | 0.00034 | 0.26 | 2.10E-09 |
| OV | **0.62** | <2.2E-16 | 0.52 | <2.2E-16 |
| PAAD |  |  | 0.25 | 0.00087 |
| PCPG | 0.33 | 6.40E-06 | 0.32 | 1.50E-05 |
| PRAD | 0.21 | 3.30E-06 | 0.28 | 3.80E-10 |
| READ | 0.46 | 5.50E-10 | **0.66** | <2.2E-16 |
| SARC | 0.28 | 5.50E-06 | 0.29 | 2.00E-06 |
| SKCM |  |  | 0.16 | 4.10E-04 |
| STAD |  |  | 0.25 | 1.50E-06 |
| TGCT |  |  | 0.43 | 3.10E-08 |
| THCA | **0.62** | <2.2E-16 | 0.37 | <2.2E-16 |
| THYM |  |  | 0.34 | 1.70E-04 |
| UCEC | 0.24 | 2.20E-08 |  |  |

**Supplementary Table 7.** Correlation analysis of *SPP1* expression with immune and stromal cells score for different tumor stages and grades (Data for presentation only P < 0.05).

| Cancer types | Immune Score | | Stromal Score | |
| --- | --- | --- | --- | --- |
|  | R | P value | R | P value |
| COAD-Stage1 | 0.42 | 0.00017 | 0.69 | <2.2e-16 |
| COAD-Stage2 | 0.57 | <2.2e-16 | 0.67 | <2.2e-16 |
| COAD-Stage3 | 0.46 | 5.8e-08 | 0.65 | <2.2e-16 |
| COAD-Stage4 | 0.5 | 3e-05 | 0.77 | <2.2e-16 |
| LGG-G2 | 0.56 | <2.2e-16 | 0.55 | <2.2e-16 |
| LGG-G3 | 0.74 | <2.2e-16 | 0.67 | <2.2e-16 |
| OV-G2 | 0.52 | 0.00034 | 0.52 | 0.00027 |
| OV-G3 | 0.65 | <2.2e-16 | 0.52 | <2.2e-16 |
| OV-Stage2 | 0.53 | 0.0098 | 0.64 | 0.0013 |
| OV-Stage3 | 0.63 | <2.2e-16 | 0.52 | <2.2e-16 |
| OV-Stage4 | 0.59 | 2.8e-06 | 0.48 | 2e-04 |
| READ-Stage1 |  |  | 0.53 | 0.0031 |
| READ-Stage2 | 0.35 | 0.012 | 0.63 | 1.6e-06 |
| READ-Stage3 | 0.52 | 0.00013 | 0.74 | <2.2e-16 |
| READ-Stage4 | 0.55 | 0.0057 | 0.62 | 0.0014 |
| THCA-Stage1 | 0.63 | <2.2e-16 | 0.37 | 1.4e-10 |
| THCA-Stage2 | 0.62 | 1.8e-06 | 0.39 | 0.0044 |
| THCA-Stage3 | 0.63 | <2.2e-16 | 0.48 | 1e-07 |
| THCA-Stage4 | 0.31 | 0.019 |  |  |

**Supplementary Table 8.** Correlation of *SPP1* expression with immunocytes in 33 cancer types.

| **Immune cell types** | **Gene** | **CancerType** | **R** | **p value** |
| --- | --- | --- | --- | --- |
| Macrophages M0 | SPP1 | BLCA | 0.22 | 7.30E-05 |
|  |  | BRCA | 0.39 | 2.20E-16 |
|  |  | COAD | 0.22 | 2.50E-06 |
|  |  | DLBC | 0.63 | 2.70E-06 |
|  |  | GBM | 0.34 | 1.20E-05 |
|  |  | HNSC | 0.2 | 7.20E-06 |
|  |  | KIRP | 0.19 | 1.10E-05 |
|  |  | LGG | 0.24 | 4.30E-06 |
|  |  | LIHC | 0.32 | 1.80E-08 |
|  |  | PRAD | 0.39 | 3.10E-15 |
|  |  | SARC | 0.37 | 3.80E-09 |
|  |  | STAD | 0.33 | 9.00E-11 |
|  |  | TGCT | 0.28 | 0.00052 |
|  |  | THCA | 0.22 | 1.20E-05 |
| Macrophages M1 | SPP1 | COAD | 0.19 | 9.30E-05 |
|  |  | GBM | -0.31 | 5.20E-05 |
|  |  | HNSC | -0.17 | 8.50E-05 |
|  |  | SARC | -0.22 | 0.00054 |
| Macrophages M2 | SPP1 | BLCA | 0.21 | 0.00017 |
|  |  | BRCA | 0.18 | 5.60E-09 |
|  |  | CESC | 0.33 | 4.30E-09 |
|  |  | COAD | 0.43 | 2.20E-16 |
|  |  | ESCA | 0.41 | 2.40E-07 |
|  |  | GBM | -0.29 | 0.00022 |
|  |  | HNSC | 0.27 | 9.90E-10 |
|  |  | LGG | -0.19 | 0.00024 |
|  |  | LUAD | 0.17 | 9.40E-05 |
|  |  | LUSC | 0.32 | 1.60E-13 |
|  |  | READ | 0.53 | 6.10E-13 |
|  |  | SARC | 0.38 | 1.20E-09 |
|  |  | STAD | 0.38 | 2.50E-14 |
|  |  | TGCT | 0.48 | 5.10E-10 |
|  |  | THYM | 0.35 | 1.00E-04 |
|  |  | UCEC | 0.22 | 1.70E-06 |
| Mast cells resting | SPP1 | ACC | -0.53 | 3.00E-04 |
|  |  | BRCA | -0.19 | 1.70E-10 |
|  |  | CESC | -0.23 | 9.60E-05 |
|  |  | GBM | -0.28 | 0.00038 |
|  |  | LIHC | -0.21 | 0.00029 |
|  |  | SARC | -0.28 | 8.50E-06 |
|  |  | STAD | -0.17 | 0.00076 |
| Mast cells activated | SPP1 | COAD | 0.19 | 8.10E-05 |
|  |  | LGG | -0.19 | 0.00034 |
|  |  | STAD | 0.19 | 0.00024 |
| Neutrophils | SPP1 | BLCA | 0.21 | 0.00011 |
|  |  | BRCA | 0.33 | 2.20E-16 |
|  |  | LIHC | 0.28 | 1.00E-06 |
|  |  | LUAD | 0.25 | 8.60E-09 |
|  |  | OV | 0.38 | 4.70E-13 |
|  |  | STAD | 0.29 | 1.20E-08 |
|  |  | UCEC | 0.27 | 4.80E-09 |
|  |  | UCS | 0.51 | 0.00096 |
|  |  | STAD | 0.29 | 1.20E-08 |
|  |  | KIRC | 0.32 | 1.40E-13 |
| B cells naïve | SPP1 | BRCA | -0.24 | 3.50E-16 |
|  |  | ESCA | -0.33 | 4.60E-05 |
|  |  | LUAD | -0.18 | 4.60E-05 |
|  |  | SARC | -0.28 | 1.10E-05 |
|  |  | TGCT | -0.56 | 9.20E-14 |
|  |  | STAD | -0.27 | 1.70E-07 |
| Dendritic cells activated | SPP1 | BRCA | 0.13 | 9.20E-06 |
|  |  | LIHC | 0.19 | 0.00088 |
|  |  | OV | 0.23 | 3.40E-05 |
|  |  | PRAD | 0.25 | 1.40E-06 |
|  |  | TGCT | 0.29 | 0.00024 |
| Plasma cells | SPP1 | BRCA | -0.2 | 1.10E-11 |
|  |  | COAD | -0.2 | 2.60E-05 |
|  |  | ESCA | -0.3 | 0.00024 |
|  |  | PRAD | -0.17 | 0.00096 |
| T cells CD4 memory activated | SPP1 | COAD | -0.26 | 4.60E-08 |
|  |  | HNSC | -0.18 | 7.80E-05 |
|  |  | THCA | 0.2 | 5.30E-05 |
| T cells CD8 | SPP1 | BRCA | -0.33 | 2.20E-16 |
|  |  | KIRP | -0.24 | 7.20E-05 |
|  |  | LUAD | -0.16 | 0.00037 |
|  |  | PRAD | -0.31 | 6.50E-10 |
|  |  | SARC | -0.3 | 2.60E-06 |
| T cells gamma delta | SPP1 | BRCA | -0.13 | 1.20E-05 |
| T cells regulatory (Tregs) | SPP1 | BLCA | -0.21 | 0.00012 |
|  |  | SKCM | -0.17 | 0.00047 |
| T cells follicular helper | SPP1 | GBM | -0.29 | 0.00021 |
|  |  | LUAD | -0.2 | 4.40E-06 |
|  |  | OV | -0.21 | 0.00017 |
| Monocytes | SPP1 | CESC | 0.24 | 2.90E-05 |
|  |  | LUAD | 0.16 | 0.00033 |
|  |  | LUSC | 0.2 | 5.00E-06 |
|  |  | TGCT | 0.31 | 8.90E-05 |
|  |  | UCEC | 0.19 | 4.20E-05 |
| Eosinophils | SPP1 | GBM | 0.28 | 0.00037 |
|  |  | LGG | 0.22 | 1.90E-05 |
|  |  | LUSC | 0.15 | 0.00082 |
|  |  | STAD | 0.24 | 3.50E-06 |
| NK cells activated | SPP1 | KIRC | -0.16 | 0.00024 |
|  |  | PCPG | -0.45 | 3.10E-05 |
|  |  | THYM | 0.37 | 3.90E-05 |
| NK cells resting | SPP1 | LIHC | -0.25 | 1.10E-05 |
